# Global Burden and Forecast of Fall‐Related Respiratory Foreign Body Aspiration in Older Adults (1990–2040): A Systematic Analysis

**DOI:** 10.1002/gch2.202500172

**Published:** 2025-11-08

**Authors:** Jian Xiao, Xiajing Liu, Wenwei Cheng, Yongquan Zhang, Yexun Song, Heqing Li

**Affiliations:** ^1^ Department of Otolaryngology‐Head and Neck Surgery The Third Xiangya Hospital of Central South University Changsha Hunan Province China; ^2^ The Third Xiangya Hospital of Central South University Changsha Hunan Province China; ^3^ Xiangya School of Public Health Central South University Changsha Hunan Province China

**Keywords:** elder, epidemiology, foreign body, global burden of disease, respiratory system

## Abstract

Foreign body in the respiratory system caused by falls (F‐RFBA) among individuals aged 70 years and older has been a growing concern globally, yet comprehensive global epidemiological data on this issue remain sparse. Data from the Global Burden of Disease (GBD) study from 1990 to 2021 are systematically reviewed to assess incidence, prevalence, and years lived with disability (YLDs) associated with F‐RFBA. Projections are made for the period up to 2040 using Bayesian age‐period‐cohort (BAPC) models. From 1990 to 2021, the incidence, prevalence, and YLDs associated with F‐RFBA among individuals aged 70 years and older showed an increasing trend. High SDI regions maintain a substantial burden, contrasting with Central Europe's decline. Global incidence is projected to increase 33.6% (2021–2040), with regional variations: decreases in Australia/New Zealand and sub‐Saharan Africa versus rises in China and the United States. The global burden of F‐RFBA among individuals aged 70 years and older has shown an increasing trend from 1990 to 2021 and is projected to rise further through 2040. Significant disparities in disease burden and trends underscore the need for targeted interventions, healthcare strengthening, and effective prevention strategies.

## Graphical abstract

Foreign body in the respiratory system is a death related medical emergency, which is often considered to be prevalent in pediatric populations. Falls are a substantial public health concern among individuals aged 70 years and older worldwide. Data about the foreign body in the respiratory system caused by falls among the elderly are lacking till now. The age‐standardized incidence rate of foreign bodies in the respiratory system caused by falls among people aged ≥ 70 worldwide increased during 1990–2021 and is predicted to increase constantly through 2040. The global burden of the foreign body in the respiratory system caused by falls among people aged ≥70 is higher in females than males across all the age subgroups, indicating that more attention should be focused on older females about this issue. The locations with high SDI values bear a significant burden of foreign bodies in the respiratory system caused by falls among people aged ≥70, which remains challenging for most of the developed countries.

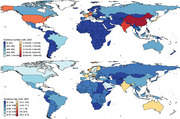

## 1 Introduction

Falls among the elderly are a prevalent and substantial public health concern, representing a leading cause of both fatal and non‐fatal injuries in this demographic [1]. While the overall burden of fall‐related injuries is widely acknowledged, specific injury mechanisms, particularly fall‐related respiratory foreign body aspiration (F‐RFBA), remain understudied despite their unique clinical severity and complex management requirements [2, 3].

Unlike general aspiration events (e.g., during meals), F‐RFBA typically occurs during a sudden fall, where the risk is heightened by the impaired protective airway reflexes common in older adults. This mechanism is distinct from typical choking incidents and is frequently complicated by concurrent injuries (e.g., fractures, head trauma), which can delay diagnosis and exacerbate outcomes [4]. Moreover, F‐RFBA is associated with high rates of respiratory failure, pneumonia, and mortality, especially in adults aged ≥70 years due to age‐related declines in physiological resilience and recovery capacity [1]. Despite its recognized severity, comprehensive global epidemiological data on F‐RFBA in the elderly remain sparse. Existing studies predominantly focus on pediatric populations, and the global burden of F‐RFBA in older adults has not been systematically examined, leaving a critical knowledge gap regarding this public health challenge [5, 6].

This study aims to address this gap by conducting a comprehensive analysis of the global burden of F‐RFBA among individuals aged 70 years and older from 1990 to 2021. Utilizing data from the Global Burden of Disease (GBD) study, we will assess the incidence, prevalence, and years lived with disability (YLDs) associated with F‐RFBA among individuals aged 70 years and older. Additionally, we stratified analyses by age group, sex, and sociodemographic index (SDI), and projected global trends from 2021 to 2040 using Bayesian age‐period‐cohort (BAPC) models.

## 2 Methods

### 2.1 Study Data

**FIGURE 1:**
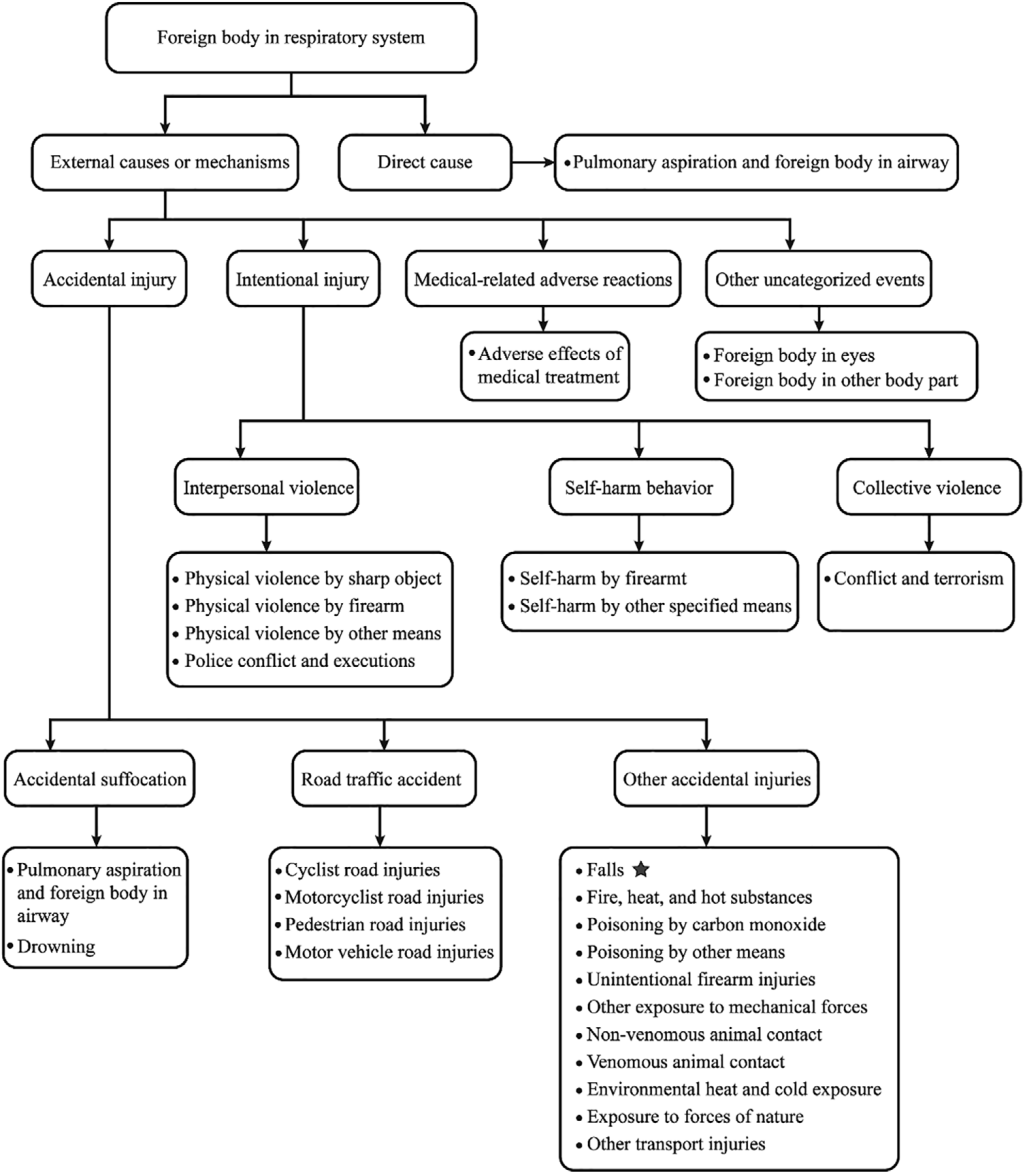
The flow diagram of a foreign body in the respiratory system caused by falls.

Our data were derived from publicly available GBD 2021 datasets [7], focusing on the incidence, prevalence, and YLD of F‐RFBA, and we extracted information on F‐RFBA. Data processing methods are detailed in our previous study [8, 9]. The ages were limited to ≥ 70 years, and divided into 70–74 years, 75–79 years, 80–84 years, 85–89 years, 90–94 years, and 95+ years. We identified fall‐related respiratory foreign body aspiration (F‐RFBA) by cross‐referencing ICD‐10 codes for foreign body inhalation (W75‐W80.9, W83‐W84.9) with fall‐related injury codes (W00‐W19.9), ensuring causality attribution. This approach distinguishes F‐RFBA from non‐fall‐related aspiration (e.g., food‐related, W78); full ICD‐10 details are in Table S4. Figure 1 shows the flow diagram of participant inclusion and exclusion for foreign bodies in the respiratory system caused by falls.

### 2.2 Pathophysiological Mechanism of F‐RFBA

#### 2.2.1 Mechanical Displacement during Impact

A fall generates sudden kinetic force that may dislodge objects held orally (e.g., food, dentures), propelling them into the airway before protective reflex activation.

#### 2.2.2 Impaired Neurological Reflexes

Falls in older adults frequently involve head trauma or transient loss of consciousness, suppressing the gag reflex and cough reflex. This neurogenic suppression creates a critical window for aspiration prior to protective reflexes recovering [10].

#### 2.2.3 Age‐Related Vulnerability

Older adults exhibit heightened susceptibility due to:

- Pre‐existing dysphagia [11];
- Delayed sensory response in the laryngopharynx [12];
- High prevalence of oral prostheses (e.g., dentures), which may detach during impact [13].

### 2.3 Statistical Analysis

Uncertainty intervals of counts and age‐standardized rates per 100 000 population were used to estimate the burden by age, sex, year, location, and socio‐demographic index (SDI). The SDI value ranges from 0 to 1. This study represented the highest education level, the highest per capita income, and the lowest fertility rate. The Bayesian age‐period‐cohort (BAPC) model was used in our study to predict the incidence rates from 2021 to 2040. Methodological details have been described elsewhere [14]. This study followed the Guidelines for Accurate and Transparent Health Estimates Reporting (GATHER) reporting guideline for cross‐sectional studies [15].

All statistics were analyzed using the R statistical software program (version 4.2.2) or SPSS 25.0 (IBM Corporation, New York, USA). P values < 0.05 were considered statistically significant.

## 3 Results

### 3.1 Global Level

**FIGURE 2:**
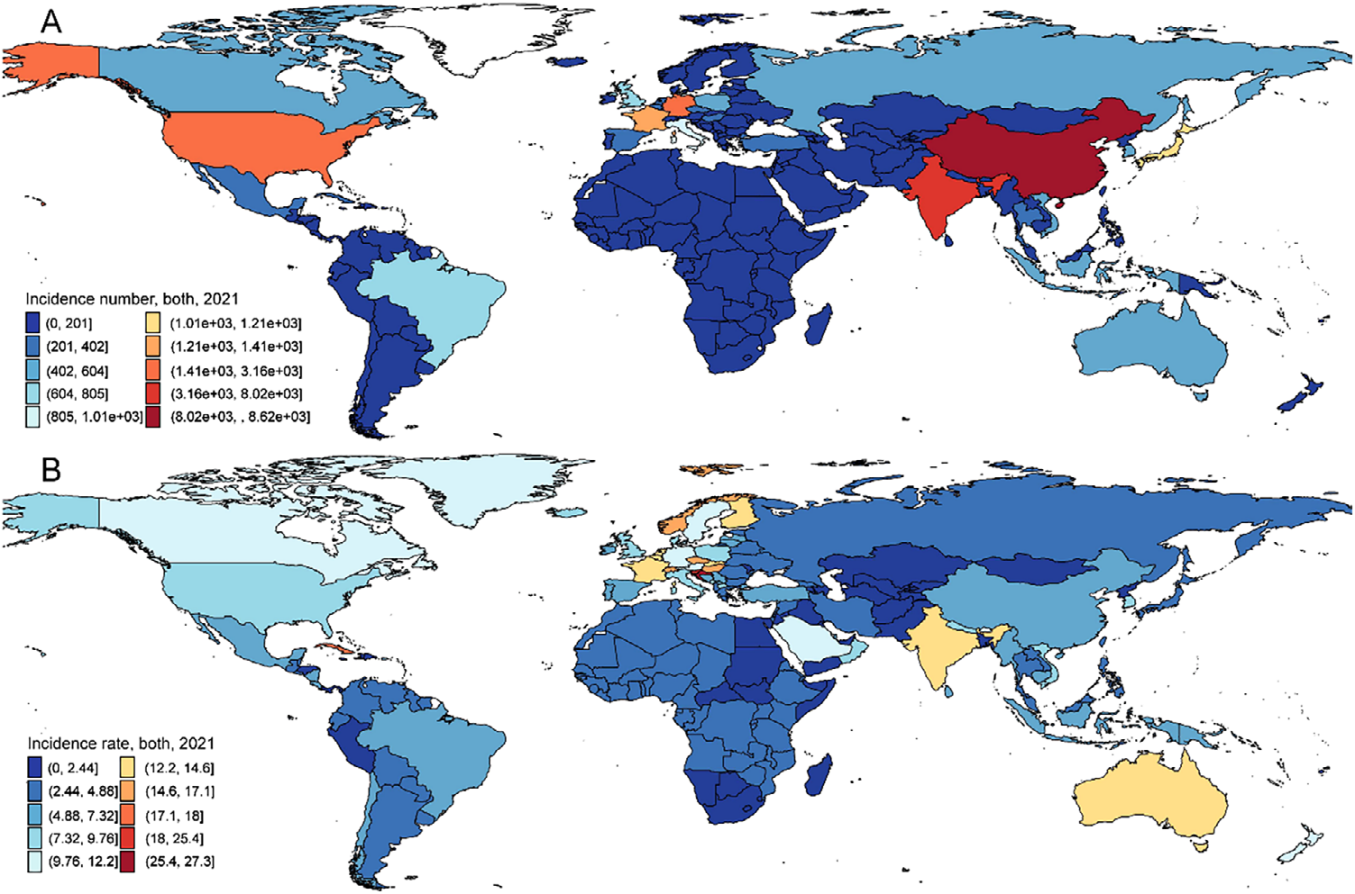
The counts and rates of incidence of foreign bodies in the respiratory system caused by falls among people aged 70 years and older for both sexes, in 2021. A counts; B rates. Rates denote age‐standardized rates.

In 2021, F‐RFBA among individuals aged 70 years and older resulted in 36,359 (95% uncertainty interval 14 097 to 79 410) new cases, 262 (95 to 607) prevalences, and 107 (38 to 248) YLDs globally. The total incidence rate increased by 20.2% (11.8% to 31.4%) from 6.1 (2.3 to 13.4) in 1990 to 7.4 (2.9 to 16.1) in 2021. The global prevalence rate rose by 21.7% (13.1% to 32%), from 0 (0 to 0.1) in 1990 to 0.1 (0 to 0.1) in 2021. Similarly, the YLD rate increased by 21.6% (13.1% to 32.6%), from 0 (0 to 0) in 1990 to 0 (0 to 0.1) in 2021 (Figure 2, Table 1; Figures S1–S5, Table S1).

**TABLE 1:** The incidence, prevalence, and YLD for foreign bodies in the respiratory system caused by falls among people aged 70 years and older in 2021 for both sexes, and percentage change of rates by GBD region, 1990–2021.

	Incidence (95%Uncertainty Interval)			prevalence (95%Uncertainty Interval)			YLD (95%Uncertainty Interval)		
	Counts	rates per 100,000 population (95% uncertainty interval)	Percentage change in rates per 100 000 population (95% uncertainty interval) (%)	Counts	rates per 100 000 population (95% uncertainty interval)	Percentage change in rates per 100 000 population (95% uncertaint interval) (%)	Counts	rates per 100 000 population (95% uncertainty interval)	Percentage change in rates per 100 000 population (95% uncertainty interval) (%)
Global	36359 (14097 to 79410)	5.6 (2.5 to 11.1)	20.2 (11.8 to 31.4)	262 (95 to 607)	0 (0 to 0.1)	21.7 (13.1 to 32)	107 (38 to 248)	0 (0 to 0.1)	21.6 (13.1 to 32.6)
Male	12242 (5349 to 24121)	8.7 (2.9 to 19.9)	36.9 (27.1 to 50.3)	88 (35 to 175)	0.1 (0 to 0.2)	37.9 (28.2 to 51)	36 (14 to 77)	0 (0 to 0)	37.9 (28.3 to 51.1)
Female	24117 (8142 to 55005)	7.4 (2.9 to 16.1)	16 (7.6 to 26.2)	174 (59 to 423)	0.1 (0 to 0.1)	17.7 (9 to 27.4)	71 (23 to 170)	0 (0 to 0.1)	17.6 (8.7 to 27.9)
Andean Latin America	83 (32 to 173)	2.5 (1 to 5.3)	57.4 (49.5 to 65.3)	1 (0 to 1)	0 (0 to 0)	58 (49.3 to 66.1)	0 (0 to 1)	0 (0 to 0)	58 (49.4 to 66.8)
Australasia	466 (166 to 1082)	12.8 (4.6 to 29.7)	49.1 (36.8 to 63.7)	3 (1 to 8)	0.1 (0 to 0.2)	50.4 (37.3 to 64.6)	1 (0 to 3)	0 (0 to 0.1)	50.4 (36.7 to 64.7)
Caribbean	278 (105 to 609)	8.7 (3.3 to 19)	50.9 (40.9 to 62)	2 (1 to 5)	0.1 (0 to 0.1)	52.3 (42.3 to 64.2)	1 (0 to 2)	0 (0 to 0.1)	52.3 (42.7 to 64.6)
Central Asia	67 (26 to 140)	2 (0.8 to 4.1)	50.7 (44.1 to 58.6)	0 (0 to 1)	0 (0 to 0)	51 (44.2 to 58.7)	0 (0 to 0)	0 (0 to 0)	50.9 (44 to 59.2)
Central Europe	1527 (580 to 3199)	10.3 (3.9 to 21.5)	−26.8 (−30.5 to −22.1)	11 (4 to 25)	0.1 (0 to 0.2)	−26.7 (−30.2 to −22.3)	5 (2 to 10)	0 (0 to 0.1)	−26.7 (−30.4 to −22.1)
Central Latin America	592 (223 to 1299)	4.3 (1.6 to 9.5)	−21.9 (−27.2 to −15.6)	4 (2 to 10)	0 (0 to 0.1)	−21.3 (−26.7 to −14.9)	2 (1 to 4)	0 (0 to 0)	−21.4 (−27 to −14.8)
Central Sub‐Saharan Africa	57 (22 to 118)	3 (1.1 to 6.3)	27.6 (14.5 to 41.2)	0 (0 to 1)	0 (0 to 0)	28.6 (15.4 to 42.2)	0 (0 to 0)	0 (0 to 0)	28.6 (15.5 to 42.6)
East Asia	8731 (3246 to 19652)	7.1 (2.6 to 15.9)	79.1 (57.5 to 112.3)	64 (23 to 141)	0.1 (0 to 0.1)	81.5 (59.6 to 115.1)	26 (9 to 58)	0 (0 to 0)	81.4 (59.4 to 115.1)
Eastern Europe	776 (289 to 1712)	3.6 (1.4 to 8)	18.8 (9.2 to 33.6)	6 (2 to 13)	0 (0 to 0.1)	18.8 (9.4 to 33)	2 (1 to 5)	0 (0 to 0)	18.7 (9.2 to 33.1)
Eastern Sub‐Saharan Africa	225 (84 to 489)	3.3 (1.2 to 7.1)	31 (20.7 to 41.9)	2 (1 to 4)	0 (0 to 0.1)	31.8 (21.4 to 42.7)	1 (0 to 2)	0 (0 to 0)	31.7 (21 to 42.5)
High‐income Asia Pacific	1583 (571 to 3717)	4.5 (1.6 to 10.6)	12 (0.2 to 29.2)	11 (4 to 26)	0 (0 to 0.1)	13 (1.2 to 30.1)	5 (2 to 11)	0 (0 to 0)	13 (0.7 to 30.2)
High‐income North America	3729 (1316 to 8956)	8.6 (3 to 20.7)	48.7 (37.7 to 64.5)	26 (9 to 63)	0.1 (0 to 0.1)	49.1 (37.8 to 64.4)	11 (4 to 25)	0 (0 to 0.1)	49 (38 to 64.9)
North Africa and the Middle East	762 (284 to 1624)	3.7 (1.4 to 8)	77.6 (65.6 to 89.9)	6 (2 to 13)	0 (0 to 0.1)	78.8 (66.5 to 91.3)	2 (1 to 5)	0 (0 to 0)	78.8 (66.2 to 91.3)
Oceania	14 (5 to 33)	5.2 (1.8 to 11.8)	54.9 (40.6 to 69.4)	0 (0 to 0)	0 (0 to 0.1)	55.9 (41.5 to 70.5)	0 (0 to 0)	0 (0 to 0)	55.9 (41.9 to 70.6)
South Asia	8298 (3093 to 18645)	11.3 (4.2 to 25.5)	17.2 (5.9 to 33.6)	61 (22 to 136)	0.1 (0 to 0.2)	17.8 (6.4 to 33.8)	25 (9 to 55)	0 (0 to 0.1)	17.7 (6 to 33.8)
Southeast Asia	1557 (579 to 3365)	5.2 (1.9 to 11.2)	35.3 (26.8 to 47.5)	11 (4 to 26)	0 (0 to 0.1)	35.7 (27.3 to 48.3)	5 (2 to 10)	0 (0 to 0)	35.7 (26.6 to 48.5)
Southern Latin America	210 (75 to 489)	3.8 (1.4 to 8.9)	11.5 (3.1 to 19.4)	1 (1 to 3)	0 (0 to 0.1)	12.1 (3.8 to 20.4)	1 (0 to 1)	0 (0 to 0)	12.1 (3.6 to 20.1)
Southern Sub‐Saharan Africa	37 (14 to 83)	1.4 (0.5 to 3.1)	−1.9 (−7.5 to 6.1)	0 (0 to 1)	0 (0 to 0)	−2 (−7.5 to 5.7)	0 (0 to 0)	0 (0 to 0)	−2 (−7.9 to 5.8)
Tropical Latin America	800 (297 to 1785)	5.6 (2.1 to 12.4)	16.2 (4.2 to 33)	6 (2 to 13)	0 (0 to 0.1)	17.2 (5.2 to 35)	2 (1 to 5)	0 (0 to 0)	17.2 (4.8 to 35.2)
Western Europe	6244 (2243 to 14585)	9.5 (3.4 to 22.2)	−1.3 (−7.5 to 5.6)	44 (15 to 104)	0.1 (0 to 0.2)	−0.6 (−7.4 to 6.3)	18 (6 to 43)	0 (0 to 0.1)	−0.6 (−7.5 to 6.3)
Western Sub‐Saharan Africa	322 (120 to 706)	4 (1.5 to 8.7)	30 (22.2 to 40)	2 (1 to 5)	0 (0 to 0.1)	30.4 (22.2 to 40.4)	1 (0 to 2)	0 (0 to 0)	30.3 (22.1 to 40.5)

YLD, years lived with disability. Rates denote age‐standardized rates.

### 3.2 Regional Level

In 2021, the largest incidence numbers were observed in East Asia (8,731, 3,246–19,652), South Asia (8,298, 3,093 to 18,645), and Western Europe (6,244, 2,243 to 14,585). Conversely, the lowest incidence numbers were reported in Oceania (14, 5 to 33), Southern Sub‐Saharan Africa (37, 14 to 83), and Central Sub‐Saharan Africa (57, 22 to 118). The highest number of F‐RFBA‐related prevalences was in East Asia, with 64 (23 to 141) cases, followed by South Asia with 61 (22 to 136) cases, and Western Europe with 44 (15 to 104) cases. In contrast, the lowest prevalence counts were in Oceania (0, 0 to 0), Southern Sub‐Saharan Africa (0, 0 to 1), and Central Sub‐Saharan Africa (0, 0 to 1). East Asia (26, 9 to 58), South Asia (25, 9 to 55), and Western Europe (18, 6 to 43) had the highest number of YLDs, while the lowest numbers were in Oceania (0, 0 to 0), Southern Sub‐Saharan Africa (0, 0 to 0), and Central Sub‐Saharan Africa (0, 0 to 0) (Table 1).

Among the 21 regions, the highest incidence rates of F‐RFBA among the elderly were observed in Australasia (12.8, 4.6 to 29.7), South Asia (11.3, 4.2 to 25.5), and Central Europe (10.3, 3.9 to 21.5). In contrast, the lowest rates were reported in Southern Sub‐Saharan Africa (1.4, 0.5 to 3.1), Central Asia (2, 0.8 to 4.1), and Andean Latin America (2.5, 1 to 5.3). The highest prevalence rates were found in Australasia (0.1, 0 to 0.2), South Asia (0.1, 0 to 0.2), and Central Europe (0.1, 0 to 0.2), while the lowest prevalence rates were in Southern Sub‐Saharan Africa, Central Asia, and Andean Latin America (all 0, 0 to 0). Although the highest YLD rates in 2021 were in Australasia, South Asia, and Central Europe (all 0, 0 to 0.1), the lowest YLD rates were in Southern Sub‐Saharan Africa, Central Asia, and Andean Latin America (all 0, 0 to 0) (Table 1).

**FIGURE 3:**
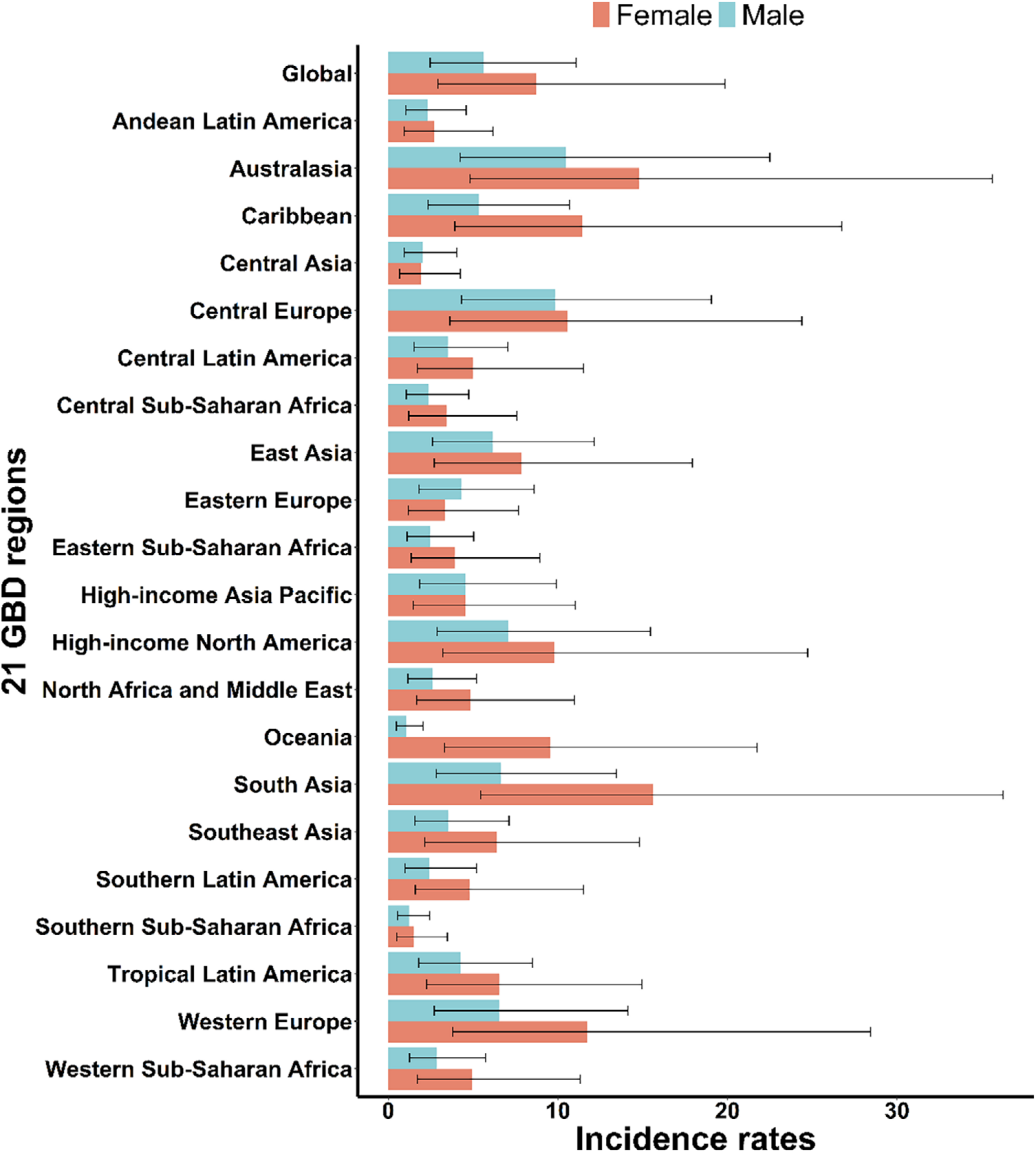
Incidence rates of F‐RFBA among people aged 70 years and older for 21 GBD regions by SDI, in 2021. Rates denote age‐standardized rates.

In 2021, incidence, prevalence, and YLD rates were higher for females than males in most of the 21 GBD regions, except Central Asia, Eastern Europe, and High‐income Asia Pacific (Figure 3, Table 1; Figures S6 and S7, Table S1).

**FIGURE 4:**
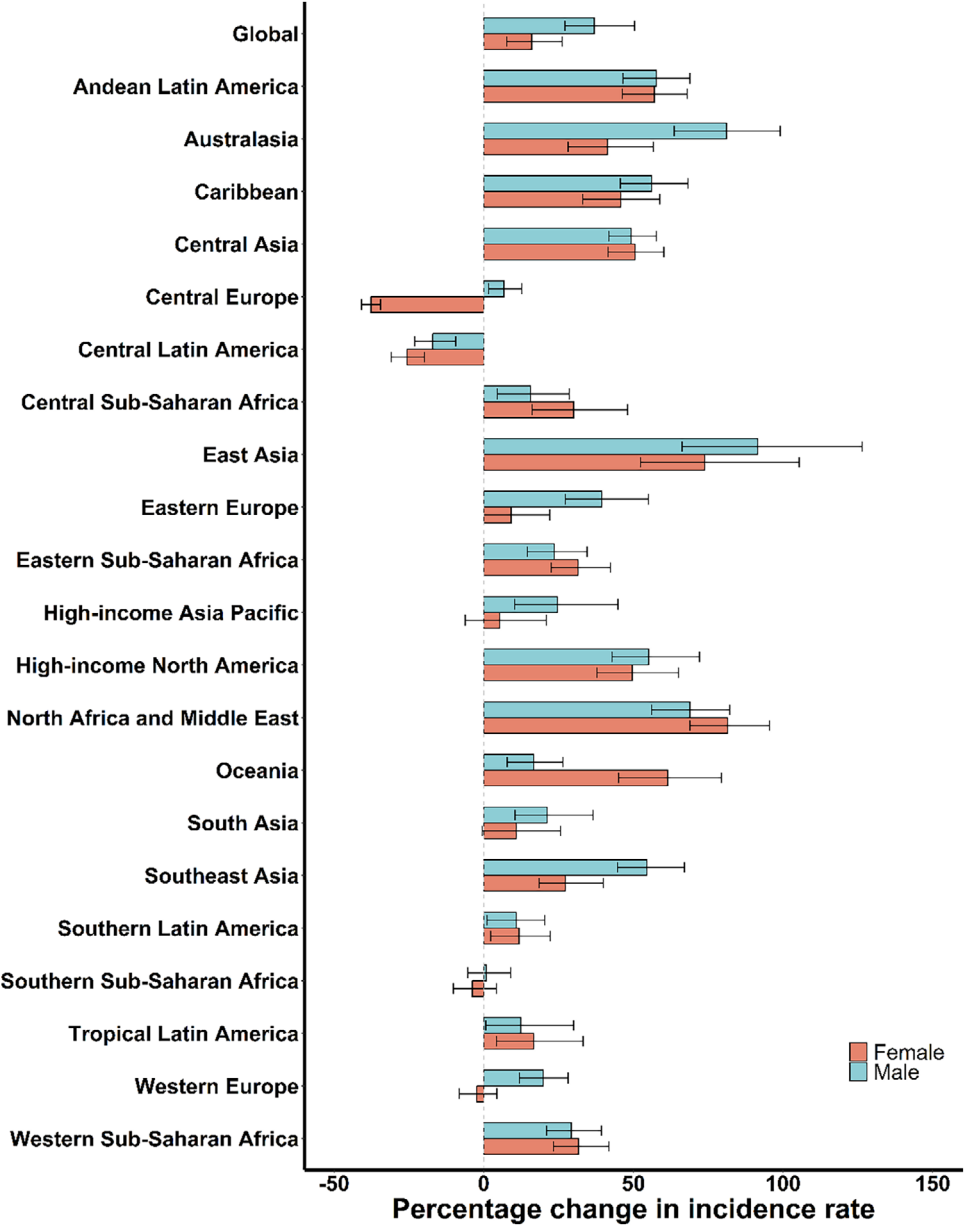
The percentage change in incidence rates of foreign bodies in the respiratory system caused by falls among people aged 70 years and older by sex for 21 GBD regions, 1990–2021. Rates denote age‐standardized rates.

Between 1990 and 2021, incidence, prevalence, and YLD rates increased globally and regionally for both males and females in most of the 21 GBD regions, excluding Central Latin America for both sexes and Central Europe, Southern Sub‐Saharan Africa, and Western Europe for females (Table 1, Figure 4; Figure S8 and S9, Table S1).

### 3.3 National Level

In 2021, China exhibited the highest incidence, prevalence, and YLDs, followed by India and the United States of America, whereas Tokelau, Niue, and Nauru had the lowest incidence, prevalence, and YLDs (Figure 2; Figures S1–S5, Tables S2 and S3).

The highest incidence rates in 2021 were observed in Croatia, Slovenia, and Andorra. Similarly, Croatia, Slovenia, and Cuba had the highest prevalence and YLD rates. Conversely, the lowest incidence, prevalence, and YLD rates were reported in Turkmenistan, South Africa, and Bangladesh (Figure 2; Figure S1–S5, Tables S2 and S3).

Between 1990 and 2021, the percentage changes in incidence, prevalence, and YLD rates varied significantly among countries. Turkey, Bhutan, and North Macedonia experienced the largest increases, while Czechia, Hungary, and Denmark showed the most substantial decreases during this period (Tables S2 and S3).

### 3.4 Age and Sex Patterns

In 2021, global numbers and rates of incidence, prevalence, and YLD were consistently higher in females than males across all age groups (Figures S10–S12).

The global incidence cases, prevalence cases, and YLDs demonstrated a downward trend with increasing age, a pattern that was consistent between males and females. The highest counts for these metrics were observed in the 70–74‐year age group. The trends in global incidence, prevalence, and YLD rates stabilized relatively with increasing age for both males and females (Figures S10–S12).

### 3.5 Burden of Foreign Body Aspiratory Caused by Falls among People Aged 70 Years and Older by SDI

**FIGURE 5:**
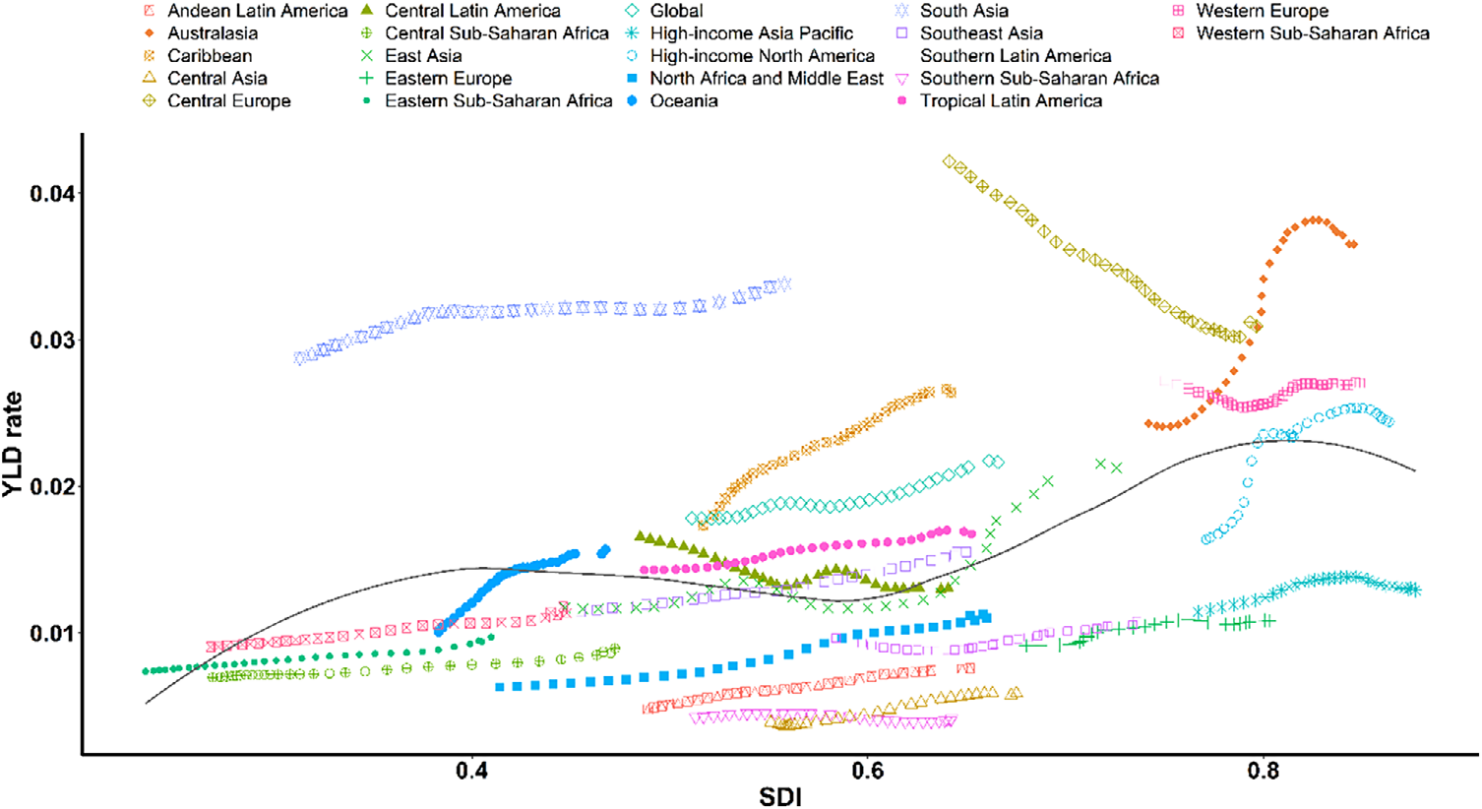
YLD rates of foreign body in the respiratory system caused by falls among people aged 70 years and older for 21 GBD regions by SDI, 1990‐2021. Rates denote age‐standardized rates.

Figure 5 depicts the trends in the YLD rate across SDI by region from 1990 to 2021. The expected pattern was nonlinear, with peaks observed at SDI values of approximately 0.8. During the study period, a majority of regions experienced an upward trend in YLD rates, with the Caribbean showing the most significant increase. In contrast, Central Europe exhibited a downward trend in YLD rates as SDI values increased from 1990 to 2021.

At the national level in 2021, the burden showed relatively stable trends by SDI values from 0 to 0.7, followed by upward trends as SDI values increased from 0.7 to 1.0. Notably, the YLD rates in countries such as Croatia, Slovenia, Andorra, Cuba, and India were significantly higher than expected. Conversely, in other countries, including Turkmenistan, Kyrgyzstan, Bangladesh, South Africa, and Azerbaijan, the YLD rates were below the expected levels based on their respective SDI values (Figure S13).

### 3.6 Prediction of Incidence Rates at Global, Regional, and National Levels from 2021 to 2040

**FIGURE 6:**
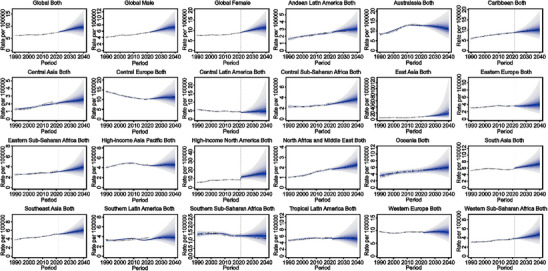
Projections of incidence rates at global and regional levels from 2021 to 2040 by BAPC models. Rates denote age‐standardized rates.

At the global level, predictions indicate an upward trend in global incidence rates for both sexes from 2021 to 2040, increasing by 33.6% from 7.4 in 2021 to 9.8 in 2040 (Figure 6).

At the regional level, Figure 6 illustrates that upward trends were predicted for incidence rates in most of the GBD regions from 2021 to 2040, except Australasia and Southern Sub‐Saharan Africa (Figure 6).

At the national level, incidence rates for 55 countries could not be predicted due to predicted absolute incidence counts lower than 1, as estimated by the BAPC model. Incidence rates for 64 countries are forecasted to decrease from 2021 to 2040, with particularly notable declines in the Republic of Moldova (−50.5%), Madagascar (−40.9%), and the Democratic People's Republic of Korea (−35.5%). Conversely, 85 countries are expected to experience increasing trends in incidence rates between 2021 and 2040, with significant increases in China (355.3%), the Syrian Arab Republic (170.4%), and the United States of America (128%) (Figure S14).

## 4 Discussion

This study presents the first comprehensive and systematic analysis of GBD data on foreign bodies in the respiratory system caused by falls (F‐RFBA) among individuals aged 70 years and older worldwide. From 1990 to 2021, the incidence, prevalence, and YLDs associated with F‐RFBA among this age group have shown an increasing trend. Projections suggest continued growth through 2040. Significant disparities by sex and region were also identified. Additionally, the elderly population in the middle‐high SDI regions bears a disproportionate burden of F‐RFBA. The current burden of F‐RFBA in this population remains substantial, with no significant improvement observed over the past decades.

Globally, the incidence of F‐RFBA among individuals aged ≥70 years increased by 20.2% from 1990 to 2021, with a corresponding rise in prevalence and YLDs. This trend contrasts with the decreasing burden of foreign body aspiration in children under 5 years of age [6, 16]. This underscores the escalating clinical burden of falls on respiratory health in aging populations. These findings highlight the critical need for targeted fall prevention programs, such as balance training, home safety assessments, and swallowing function screenings, particularly in denture wearers, to mitigate F‐RFBA risk among older adults. The observed increase in incidence, prevalence, and YLDs indicates that falls are becoming an increasingly prevalent and severe issue among older adults. This trend necessitates urgent intervention by healthcare systems and policymakers.

The burden of F‐RFBA among the elderly varies across SDI levels, regions, and countries. Despite public health achievements disproportionately benefiting high SDI regions, areas such as Australasia, Western Europe, and High‐income North America continue to bear a significant burden of F‐RFBA in the elderly population. Notably, this burden has exhibited an upward trend over the past two decades. This contrasts with the declining trend observed in respiratory foreign body aspiration among children under 5 years old [6, 17, 18, 19]. This phenomenon can be attributed to three key factors: population aging, an elevated risk of falls among the elderly, and robust health management systems in high SDI regions. These systems enable more effective monitoring and recording of respiratory foreign body aspiration cases [20, 21, 22, 23]. These results are consistent with studies from several high‐income countries reporting increased healthcare encounters related to fall‐related aspiration in the elderly, yet contrast with reports from regions with younger demographics where such incidents remain rare [24, 25, 26]. These findings suggest that policymakers in high SDI regions should refine existing policies to deliver higher‐quality, more effective healthcare services, thereby alleviating the burden of F‐RFBA among the elderly.

Notably, Central Europe, a middle SDI region, demonstrated declining burden trends over the past two decades. This is consistent with decreasing hip fracture rates from falls, which may be related to improvements in disease management in the region [27, 28, 29]. In low, low‐middle, and middle SDI regions of Asia and Africa, the burden of F‐RFBA among the elderly remains comparatively low except in South Asia. This can be attributed to a combination of factors, including limited medical resources, inadequate health management systems, and insufficient social attention and policy support [30, 31, 32]. Furthermore, biological factors such as higher rates of sarcopenia and osteoporosis in certain populations, along with social determinants like access to preventive care and public health awareness, may contribute to these regional disparities [26]. Additionally, it is important to note that in 2021, China recorded the highest incidence, prevalence, and YLDs of F‐RFBA among individuals aged ≥70 years. India and the United States of America followed findings partially attributable to their large aging populations [33].

Our findings align with the majority of literature reports from various countries on sex‐specific fall risk patterns among older adults [34, 35, 36]. While the global numbers and rates of incidence, prevalence, and YLDs were higher in females aged ≥70 years compared to age‐matched men, it should be noted that males generally exhibit higher age‐standardized fall rates across broader population studies [37]. This paradoxical age‐dependent sex disparity may be attributed to multifactorial mechanisms: (1) behavioral determinants: males exhibit greater exposure to fall hazards through occupational risks (e.g., construction, mining) and risk‐taking behaviors, including alcohol consumption, particularly in younger age cohorts [38, 39]. (2) Biological factors: postmenopausal women experience accelerated bone mineral density loss due to estrogen deficiency, leading to higher osteoporosis prevalence [40]. Concurrently, age‐related sarcopenia and reduced physical activity further compromise musculoskeletal integrity [41]. (3) psychosocial components: cross‐cultural studies consistently report a higher prevalence of depression and psychological distress among elderly women. Such affective disorders have been independently associated with fall risk through impaired balance awareness and psychotropic medication use. This may potentially exacerbate fall‐related respiratory foreign body aspiration [42, 43]. To address the gender differences in the burden of F‐RFBA among older adults, it is imperative to design and implement targeted interventions. These strategies should be adapted to variations in physical function and psycho‐emotional profiles, and may include community‐based fall prevention initiatives, routine denture safety assessments, and dysphagia screening for high‐risk populations‐particularly women. Such targeted interventions are designed to reduce the health risks and alleviate the disease burden associated with respiratory foreign body aspiration in this demographic, thereby contributing to improved global public health outcomes.

Predicted trends in global F‐RFBA incidence reveal a complex and multifaceted landscape, with marked variations observed at both regional and national levels. Globally, from 2021 to 2040, the incidence is projected to rise by 33.6%. This concerning increase underscores the necessity for enhanced preventive measures and public health interventions to mitigate the risk factors associated with foreign body aspiration, which aligns with global aging trends [44]. It also emphasizes the need for integrative geriatric care that includes swallowing assessments and environmental modifications to prevent falls. At the regional level, Australia and New Zealand, as well as sub‐Saharan Africa, are expected to experience a decline in the incidence of fall‐induced foreign body aspiration among the elderly. 64 countries‐including the Republic of Moldova, Madagascar, and the Democratic People's Republic of Korea‐are projected to see significant reductions in incidence rates. Conversely, 85 countries‐such as China, the Syrian Arab Republic, and the United States of America‐are anticipated to face rising incidence rates. This divergence may stem from targeted public health interventions, improved healthcare infrastructure, and heightened awareness and preventive strategies in certain regions and countries [45, 46]. Conversely, the absence of such interventions in other regions may drive the projected increases.

This study has several limitations. First, the completeness and accuracy of data differ across different countries and regions, potentially introducing biases into the GBD reporting system. A significant concern is the potential for misclassification bias, as not all aspiration events that are clinically coded following a fall may be directly and solely caused by the fall itself. Conversely, under‐reporting is a major issue, particularly in low‐ and middle‐income countries, where surveillance systems may be less developed; this likely leads to a substantial underestimation of the true burden of F‐RFBA in these regions. Second, we did not analyze which risk factors predominate or assess the annual changes in the contributions of these risk factors, thereby limiting our ability to provide more targeted recommendations. Furthermore, our analysis is based on GBD secondary data, which, while comprehensive, limits our capacity to establish causal inferences or explore individual‐level risk factors in greater depth. Finally, even in countries with high SDI, official data for 2021 might be delayed, as the GBD employs an iterative estimation framework to provide estimates over a period; the official 2021 data will be updated once available and will be reflected in future iterations of the GBD.

## 5 Conclusion

From 1990 to 2021, the global burden of F‐RFBA among individuals aged 70 years and older has shown an increasing trend, and this upward trajectory is projected to continue from 2021 to 2040. Significant disparities in burden levels and temporal trends are evident across various regions, countries, and gender groups. These findings highlight the necessity for targeted public health interventions, improved healthcare infrastructure, and enhanced awareness and preventive strategies, particularly in regions and countries with the highest burden.

## Funding

This work was supported by grants from the National Science Foundation of Hunan Province (2023JJ30840) and the Wisdom Accumulation and Talent Cultivation Project of the Third Xiangya Hospital of Central South University (YX202210).

## Ethics Statement

The institutional review board of the Third Xiangya Hospital of Central South University in Hunan Province, China, determined that the study did not need approval because it used publicly available data because it used publicly available data.

## Conflicts of Interest

The authors declare no conflicts of interest.

## Supporting information




**Supporting File**: gch270065‐sup‐0001‐TableS1‐S4.docx

## Data Availability

These data were derived from the following resources available in the public domain: http://ghdx.healthdata.org/gbd‐results‐tool.
