# Supplementary material for: Global Burden and Forecast of Fall‐Related Respiratory Foreign Body Aspiration in Older Adults (1990–2040): A Systematic Analysis
Source: Glob Chall. 2025 Nov 8;9(12):e00172. doi: 10.1002/gch2.202500172 (PMC12697084; doi:10.1002/gch2.202500172)
Supplement: Supplementary file 1 — Supporting File: gch270065‐sup‐0001‐TableS1‐S4.docx [file GCH2-9-e00172-s001.docx]

**Figure S1** Prevalence counts of foreign body in respiratory system caused by falls among people aged 70 years and older for both sexes, in 2021.

**
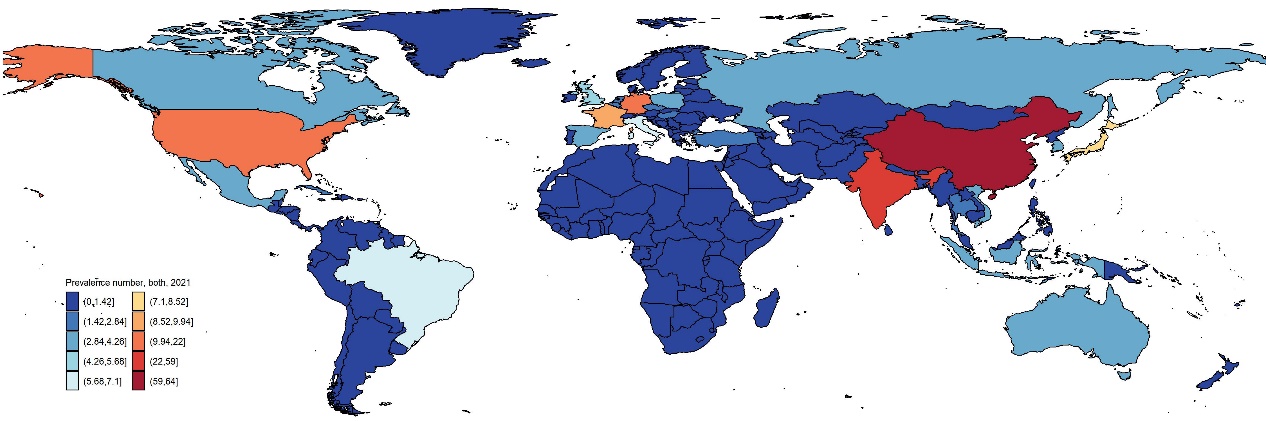
**

**Figure S2** YLD counts of foreign body in respiratory system caused by falls among people aged 70 years and older for both sexes, in 2021.

**
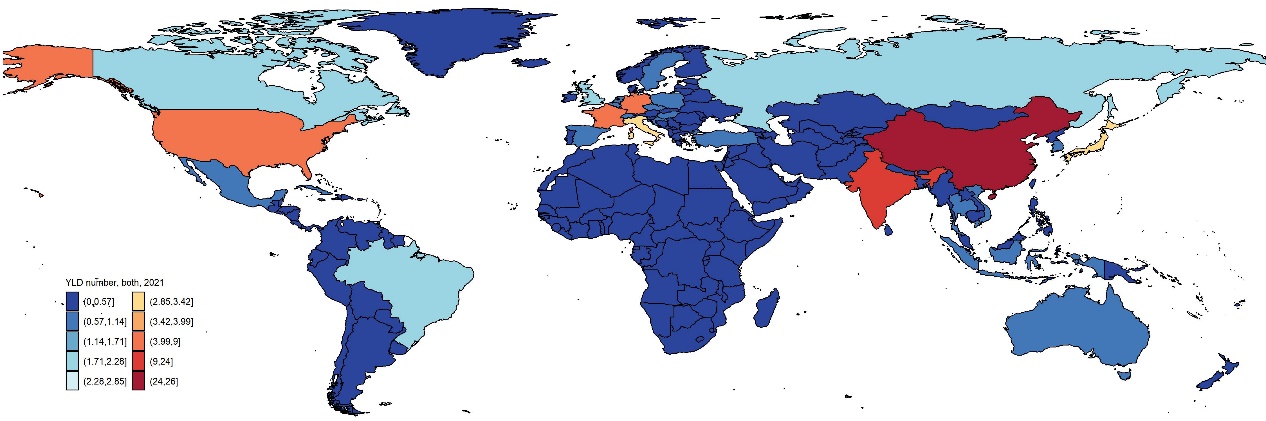
**

**Figure S3** Prevalence rates of foreign body in respiratory system caused by falls among people aged 70 years and older for both sexes, in 2021.

**
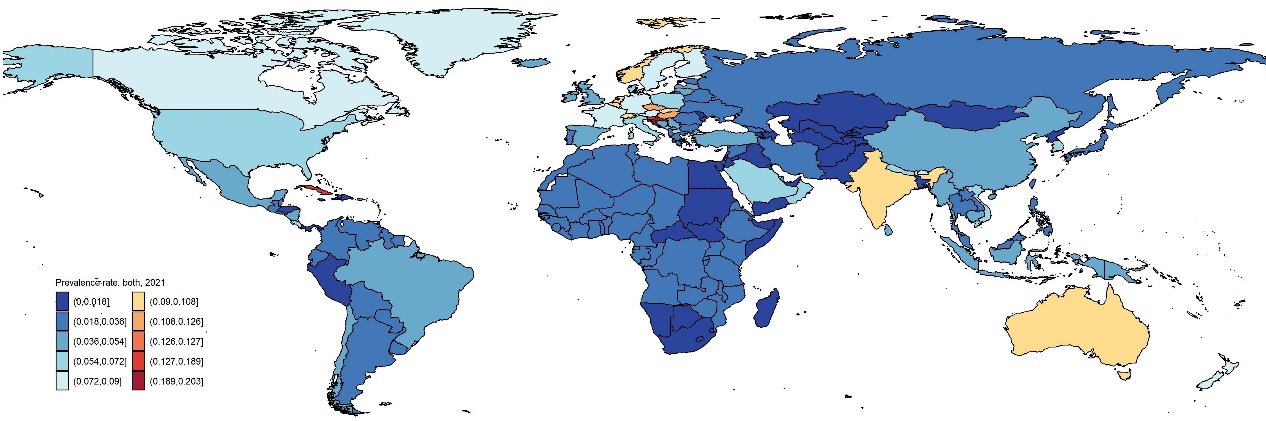
**

Rates denote age-standardized rates.

**Figure S4** YLD rates of foreign body in respiratory system caused by falls among people aged 70 years and older for both sexes, in 2021.

**
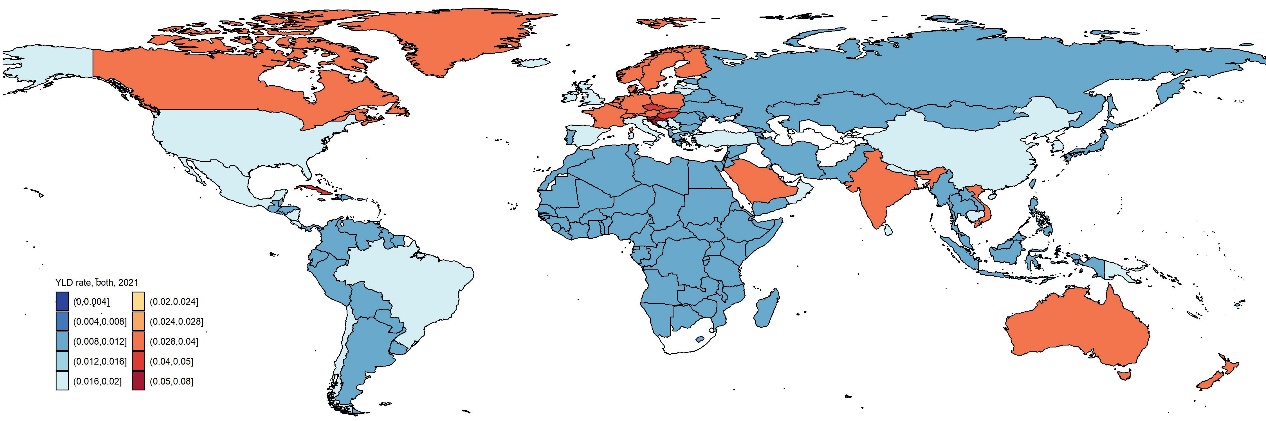
**

Rates denote age-standardized rates.

**Figure S5.** The 204 countries and territories distribution by SDI quintile, 2021.


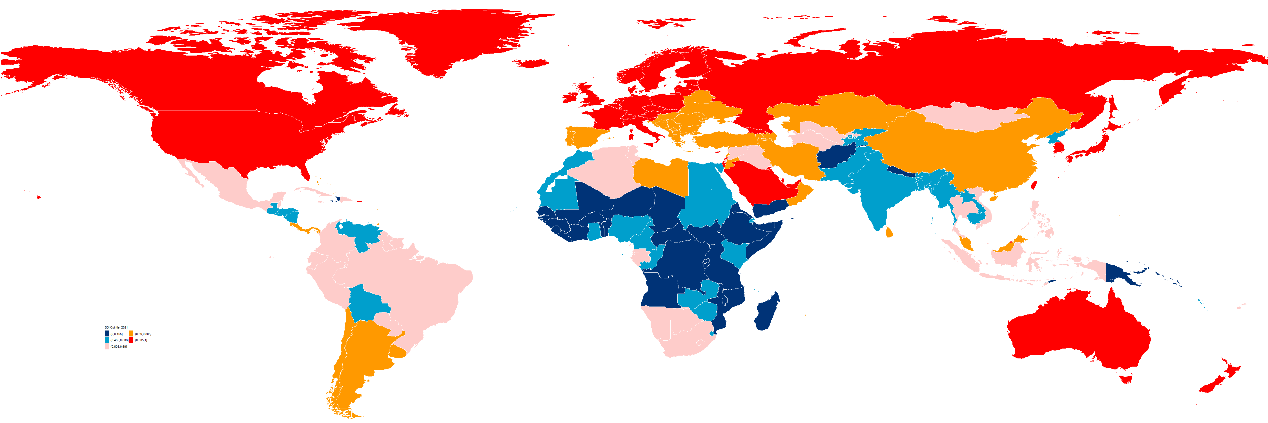


SDI, Socio-Demographic Index.

**Figure S6.** Prevalence rates of foreign body in respiratory system caused by falls among people aged 70 years and older for 21 Global Burden Disease regions by sex, 2021.

**
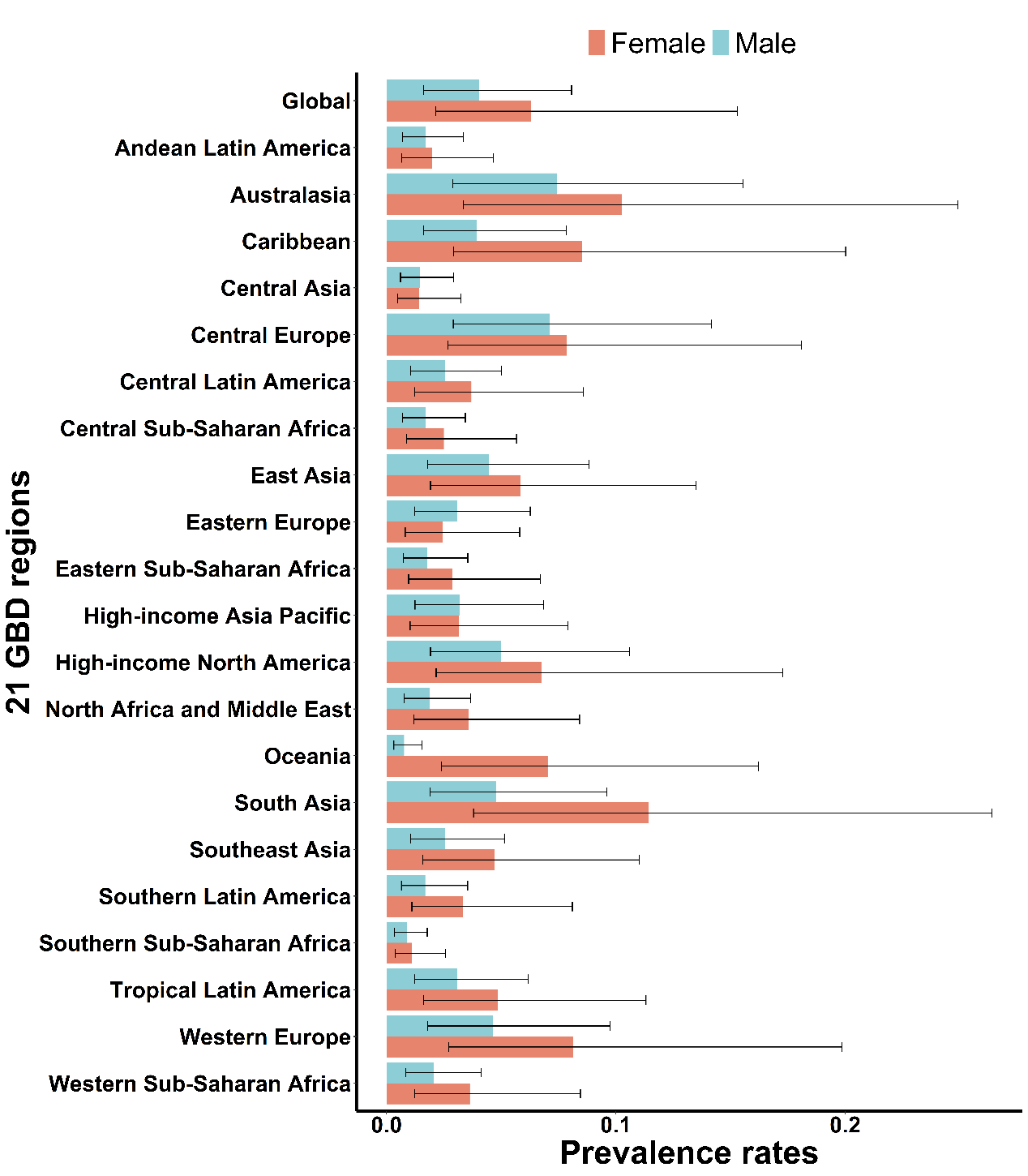
**

Rates denote age-standardized rates.

**Figure S7.** YLD rates of foreign body in respiratory system caused by falls among people aged 70 years and older for 21 Global Burden Disease regions by sex, 2021.

**
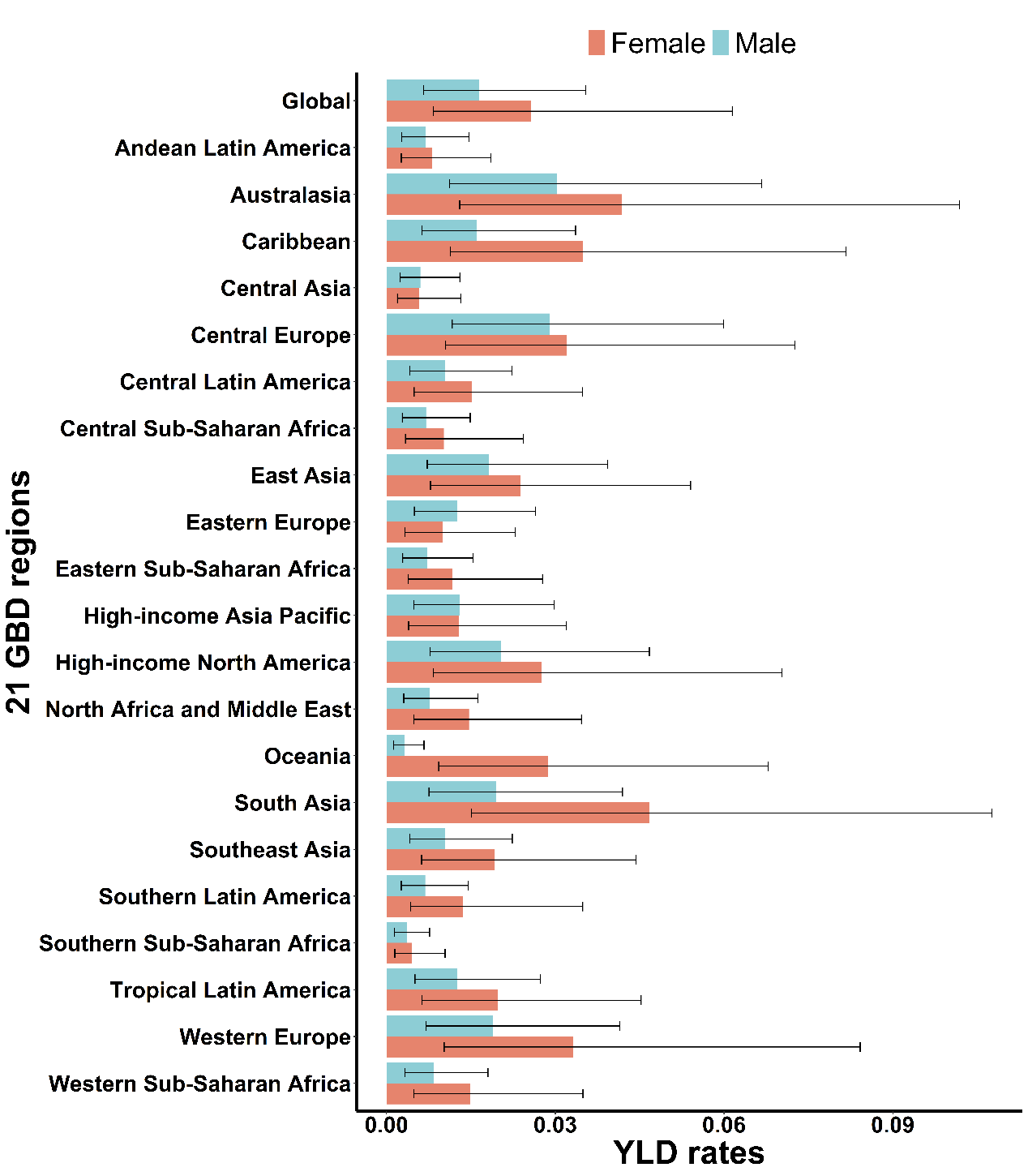
**

Rates denote age-standardized rates.

**Figure S8.** The percentage change in prevalence rate of foreign body in respiratory system caused by falls among people aged 70 years and older by sex for 21 Global Burden of Disease regions, 1990-2021.

**
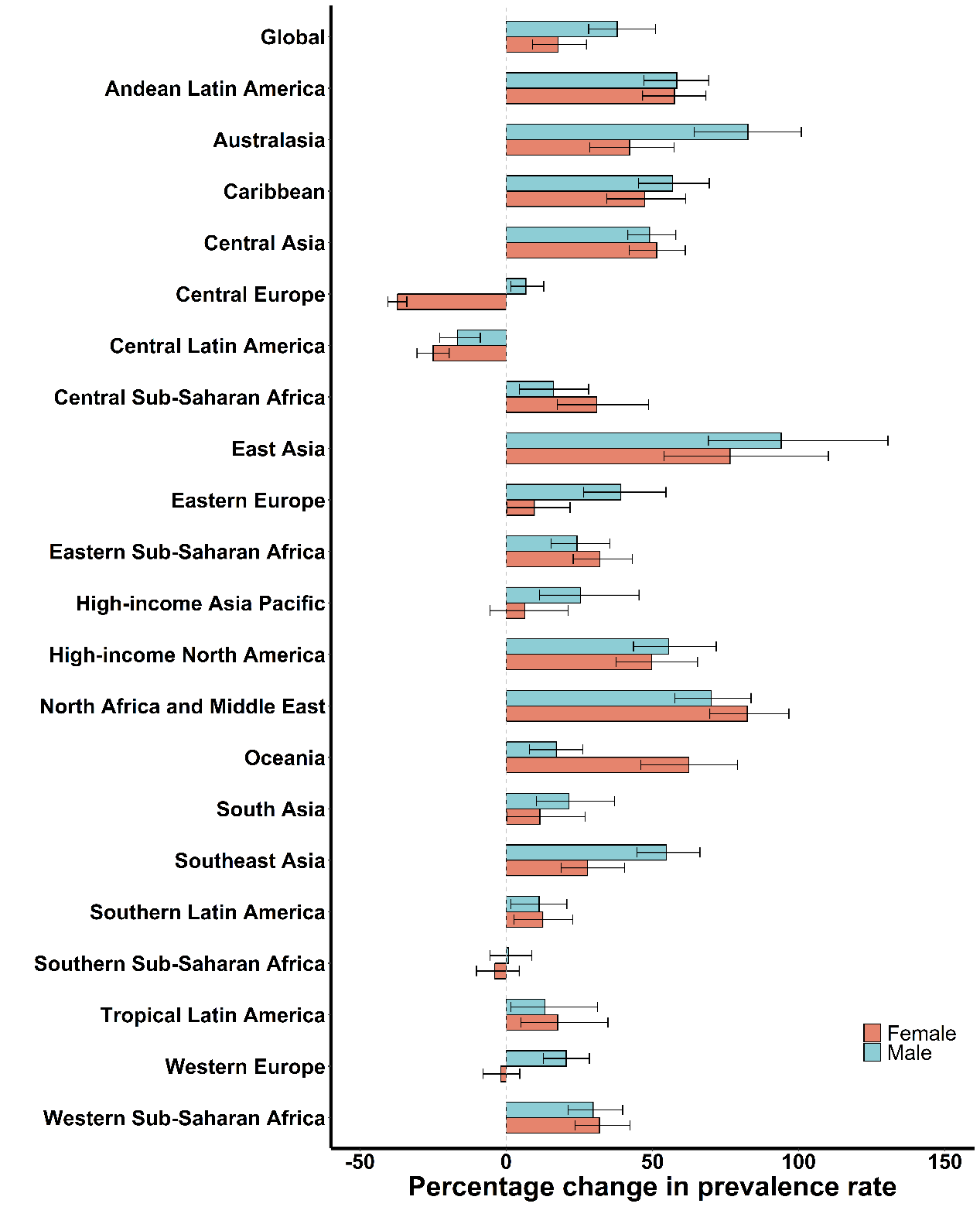
**

Error bars indicate the 95% uncertainty intervals (95% UI) for prevalence.

Rates denote age-standardized rates.

**Figure S9.** The percentage change in YLD rate of foreign body in respiratory system caused by falls among people aged 70 years and older by sex for 21 Global Burden of Disease regions, 1990-2021.

**
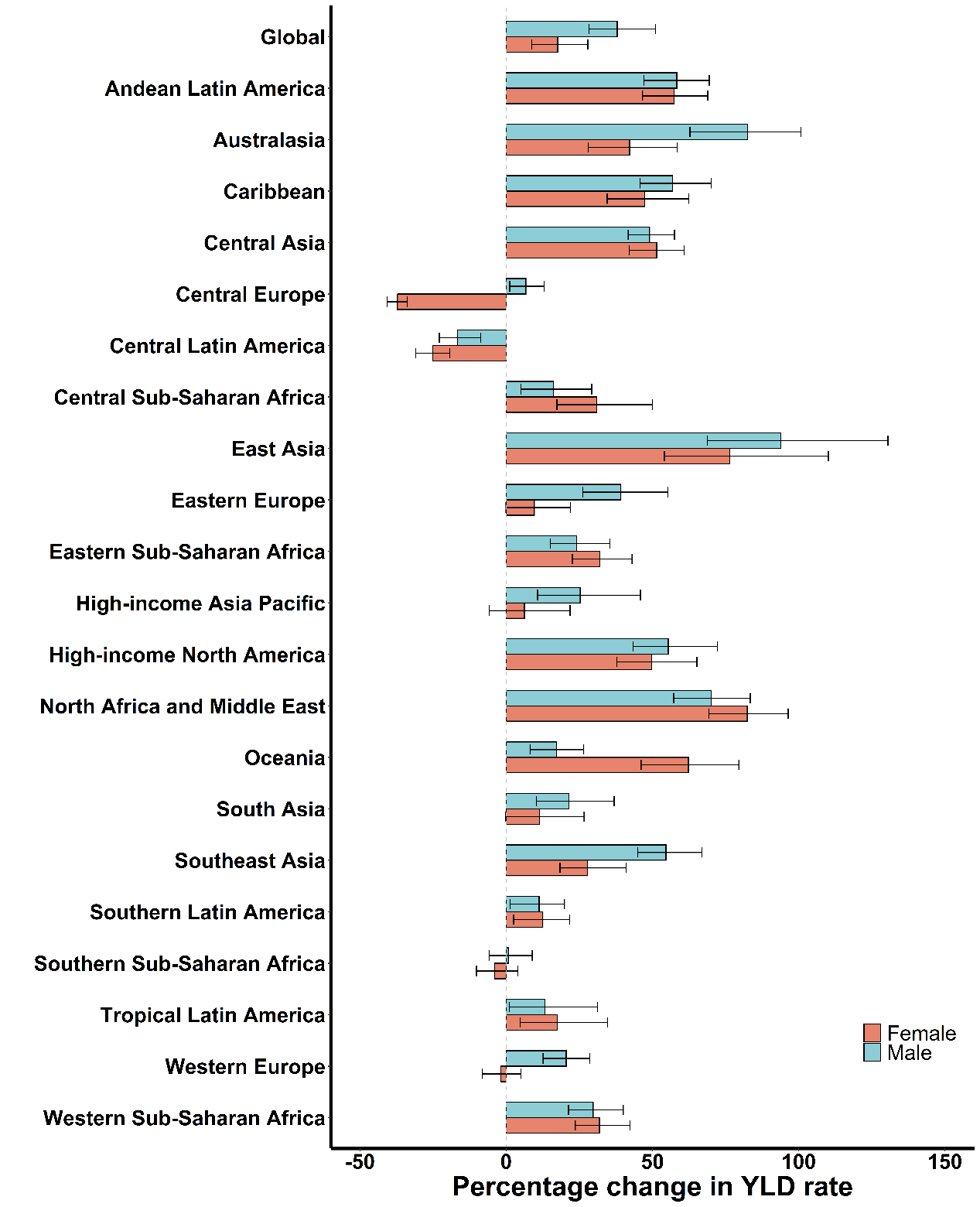
**

Error bars indicate the 95% uncertainty intervals (95% UI) for YLD. Rates denote age-standardized rates.

**Figure S10.** Global incidence counts and rates of foreign body in respiratory system caused by falls among people aged 70 years and older by age and sex, 2021.

**
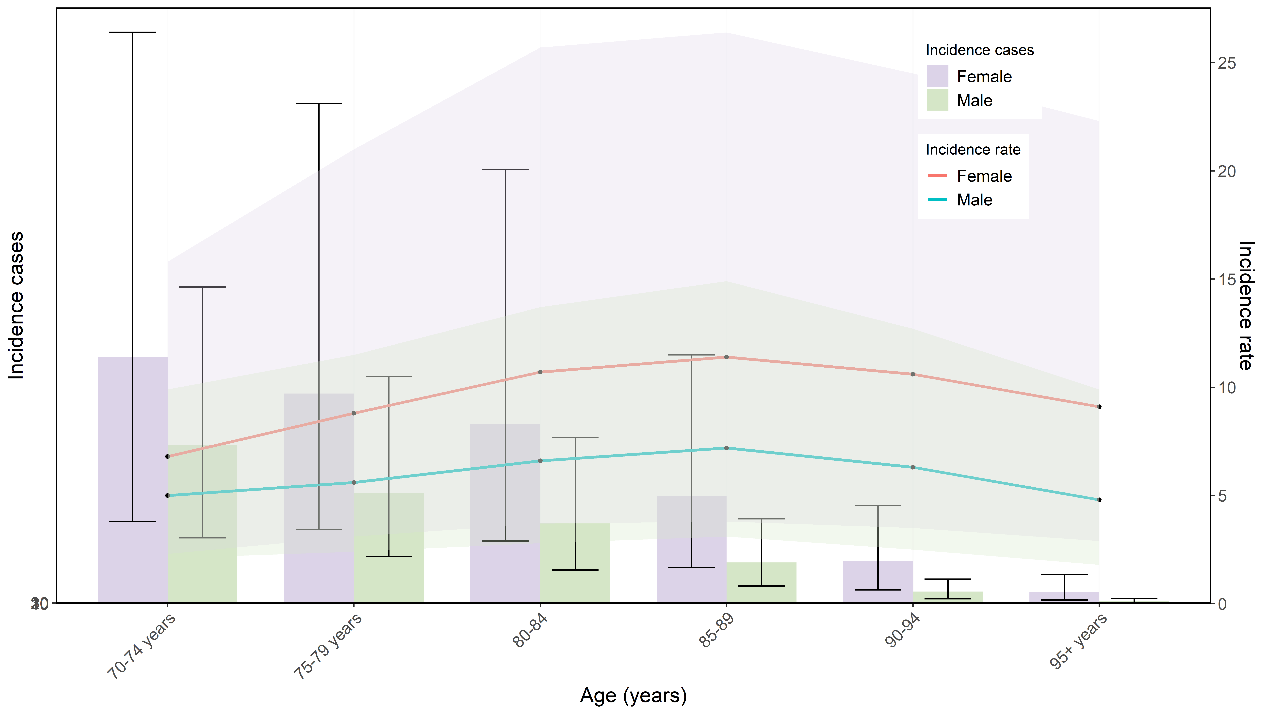
**

Error bars indicate the 95% uncertainty intervals (95% UI) for incidence. Shading indicates the upper and lower limits of the 95% UI. Rates denote age-standardized rates.

**Figure S11.** Global prevalence counts and rates of foreign body in respiratory system caused by falls among people aged 70 years and older by age and sex, 2021.

**
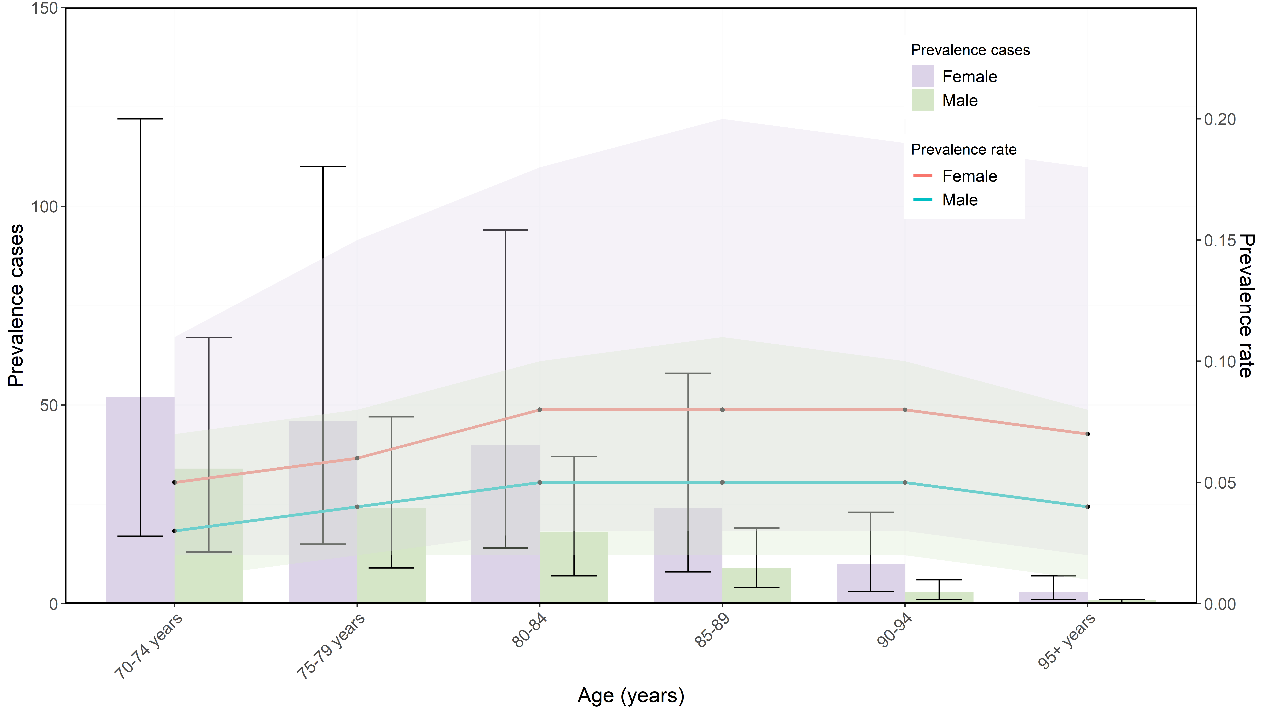
**

Error bars indicate the 95% uncertainty intervals (95% UI) for prevalence. Shading indicates the upper and lower limits of the 95% UI. Rates denote age-standardized rates.

**Figure S12.** Global YLD counts and rates of foreign body in respiratory system caused by falls among people aged 70 years and older by age and sex, 2021.

**
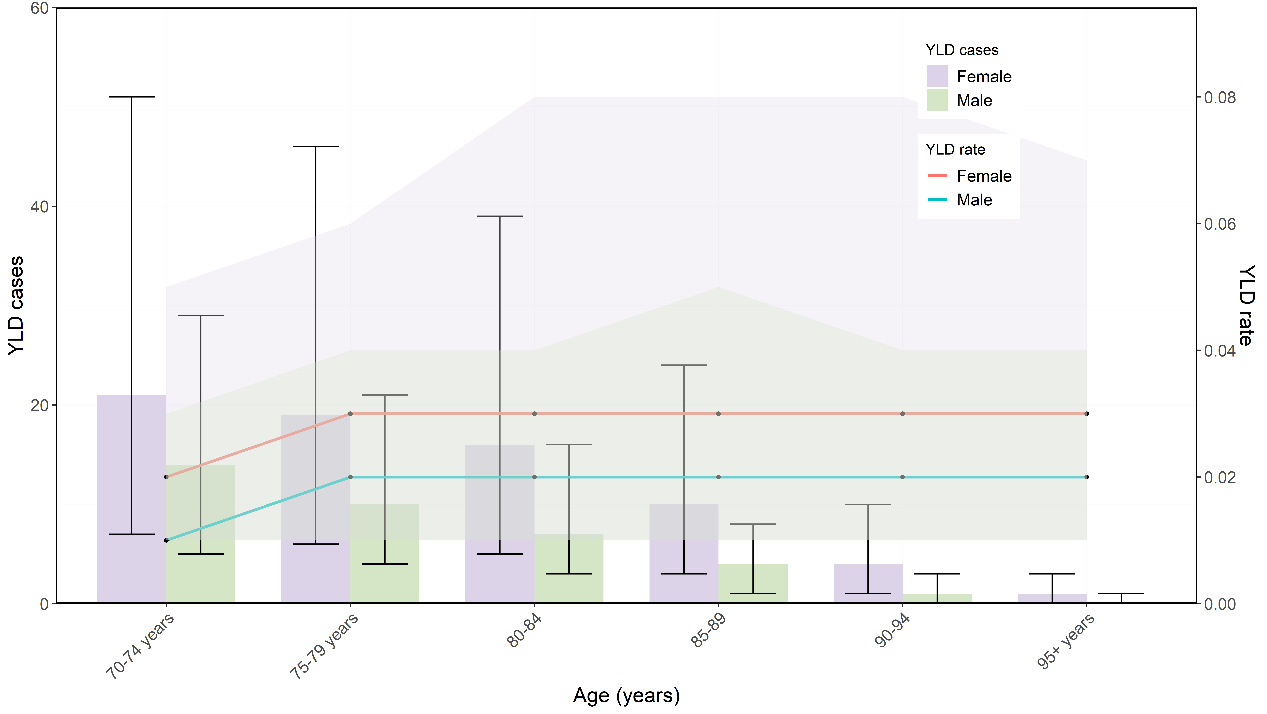
**

Error bars indicate the 95% uncertainty intervals (95% UI) for YLD. Shading indicates the upper and lower limits of the 95% UI. Rates denote age-standardized rates.

**Figure S13.** YLD rates of foreign body in respiratory system caused by falls among people aged 70 years and older for 204 countries and territories by SDI, 2021.


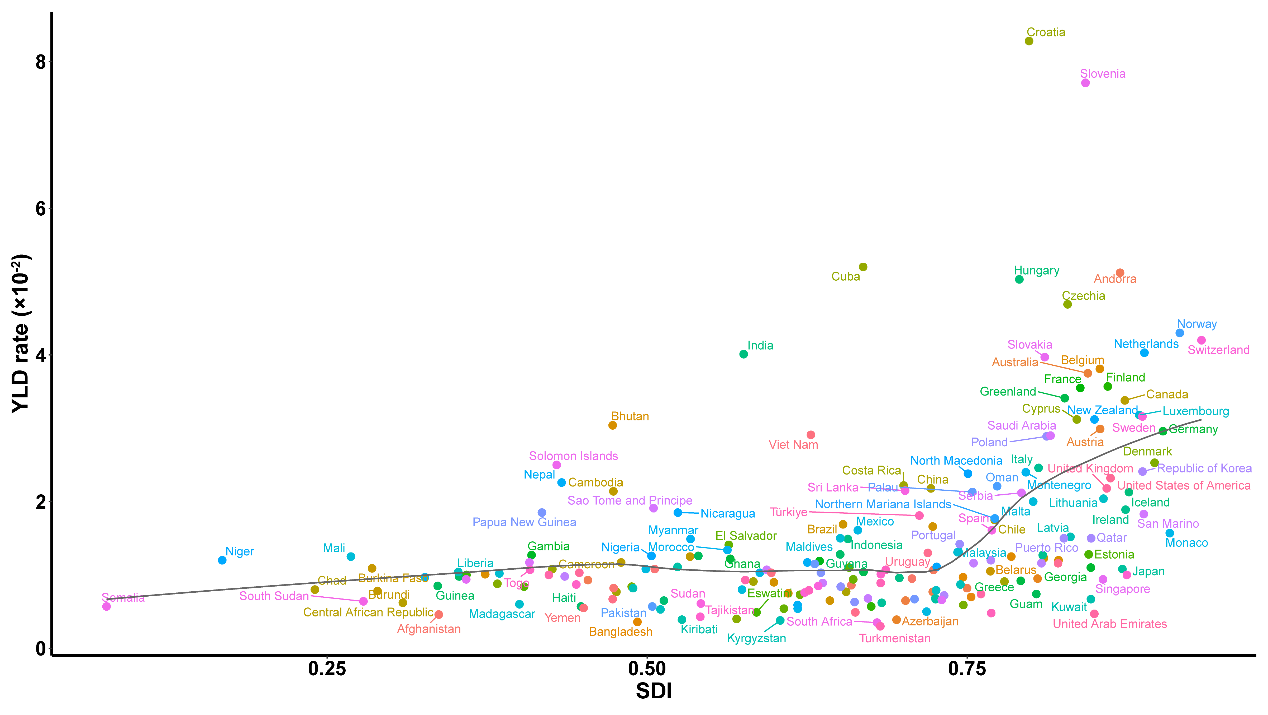


Black line represents the expected YLD rates of foreign body in respiratory system caused by falls among people aged 70 years and older based on SDI.

SDI, Socio-demographic Index. YLDs, years lived with disability. Rates denote age-standardized rates.

**Figure S14.** Trends in incidence rates of 149 countries and territories from 1990 to 2040 for both sexes by BAPC model.


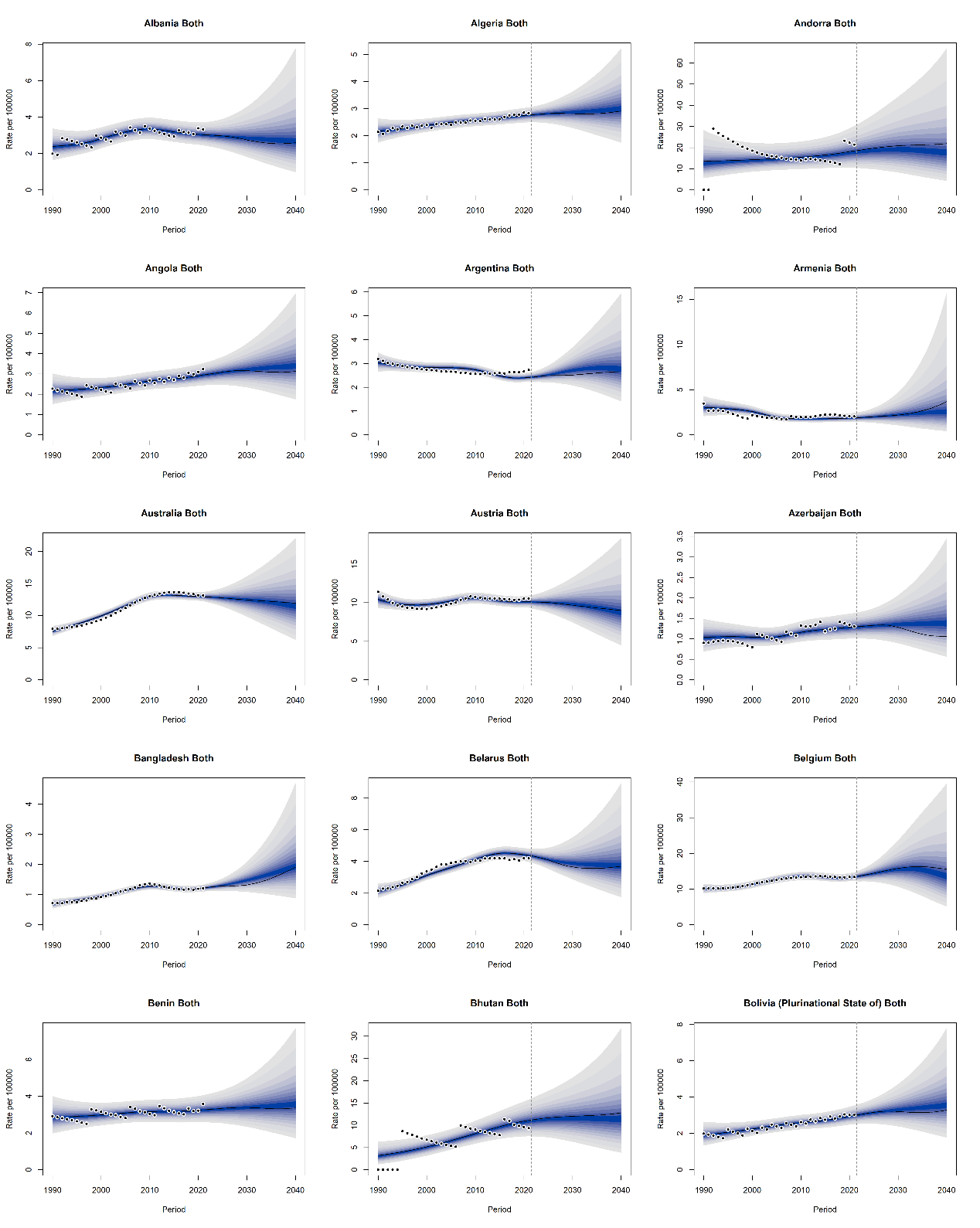


**
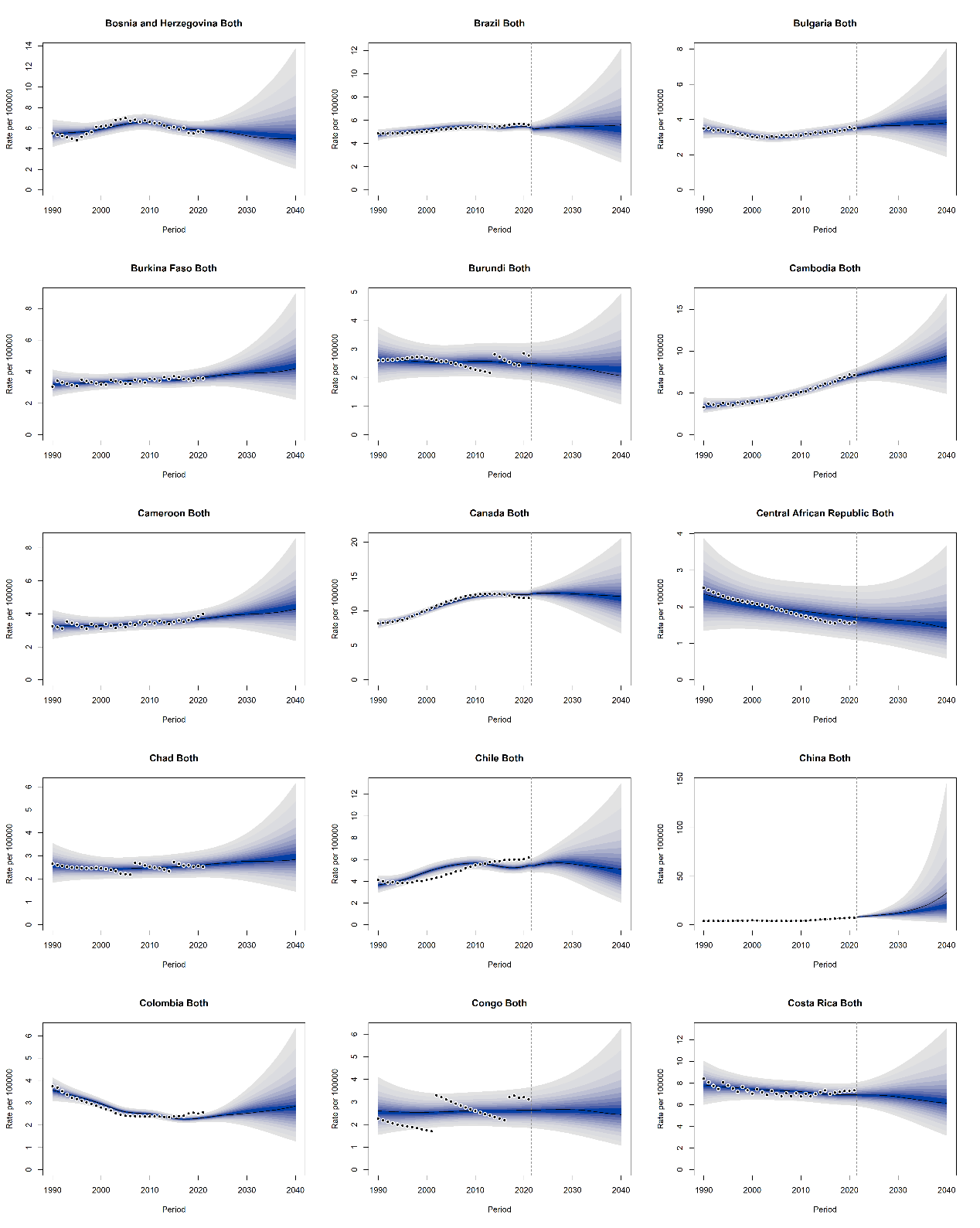
**

**
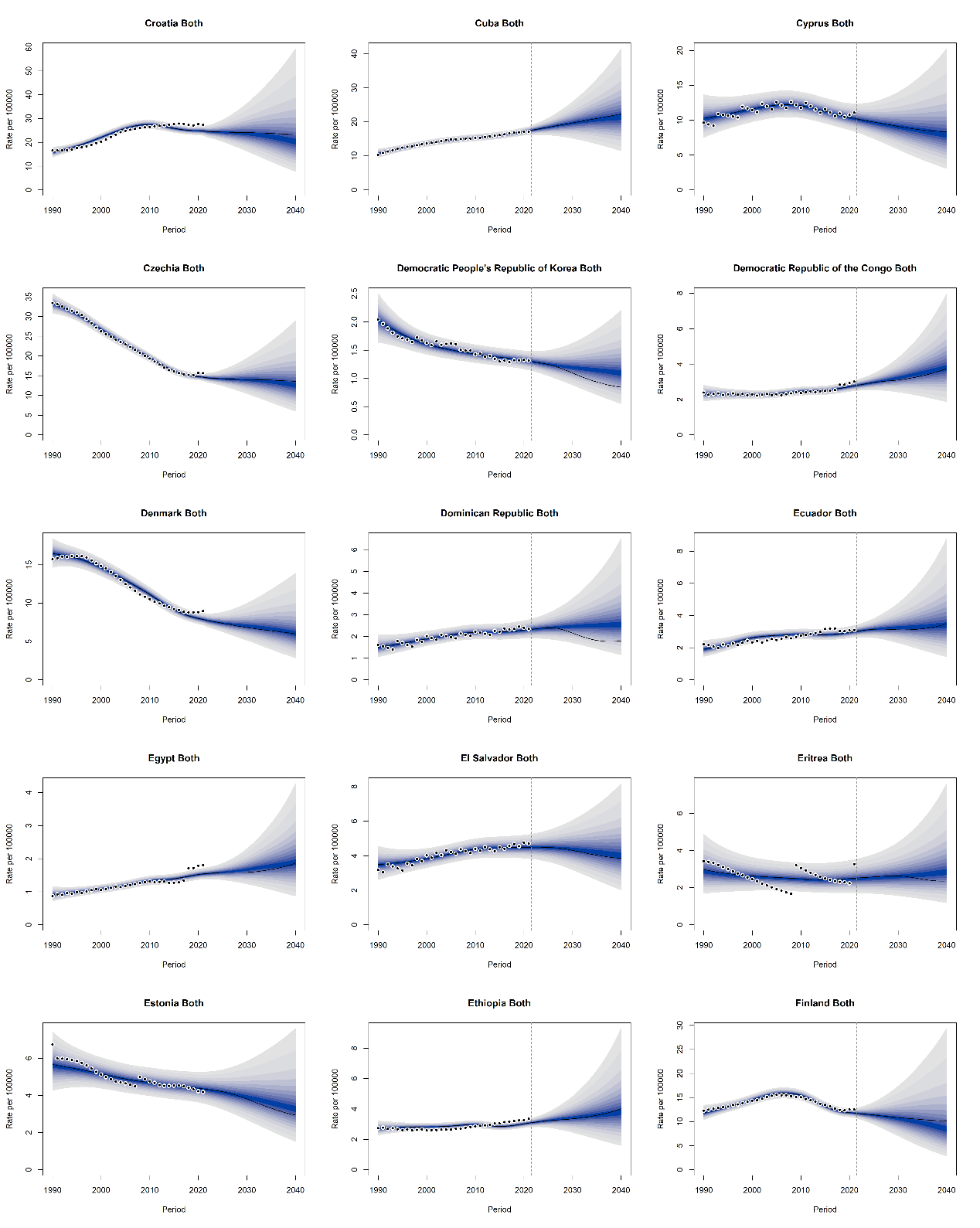
**

**
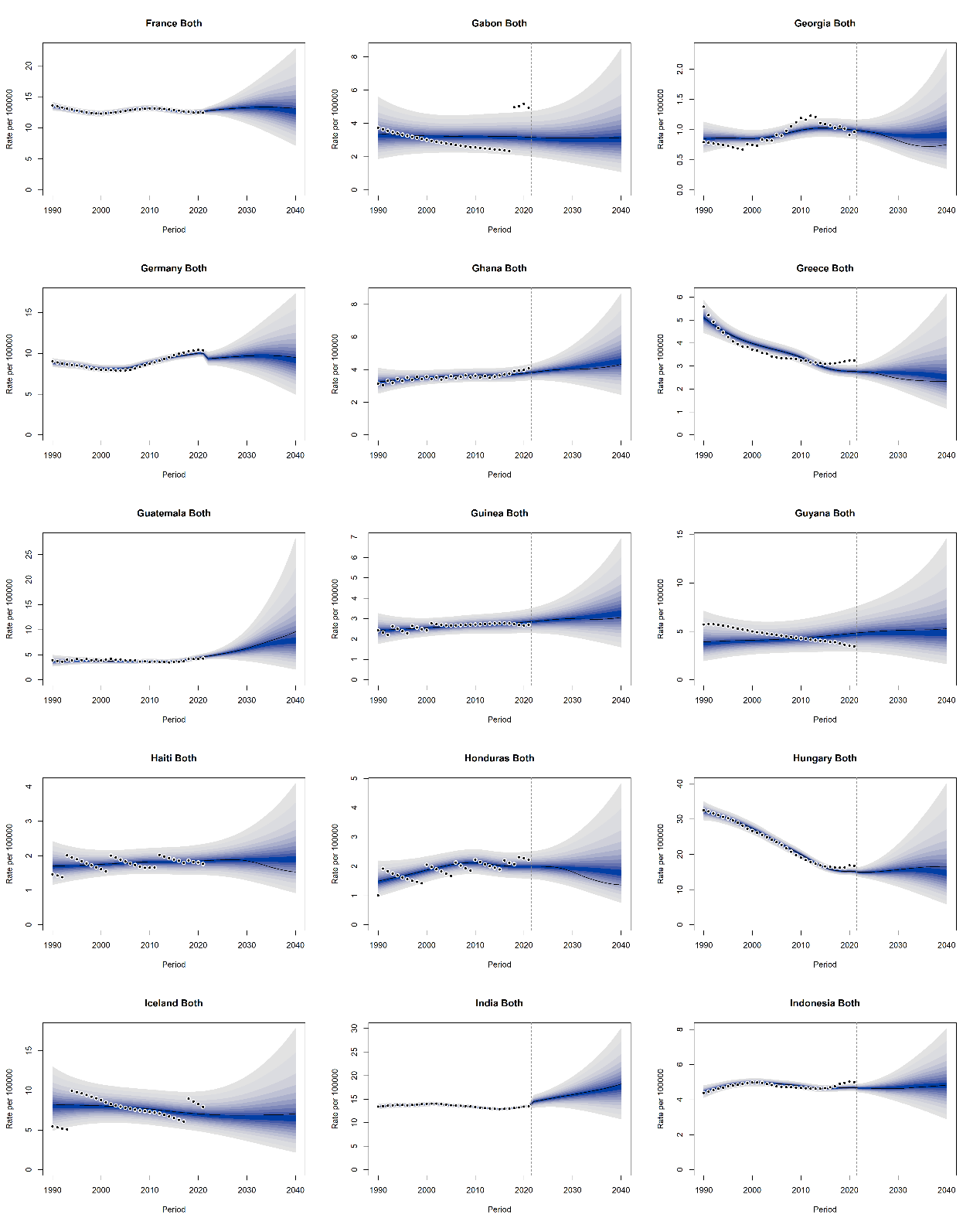
**

**
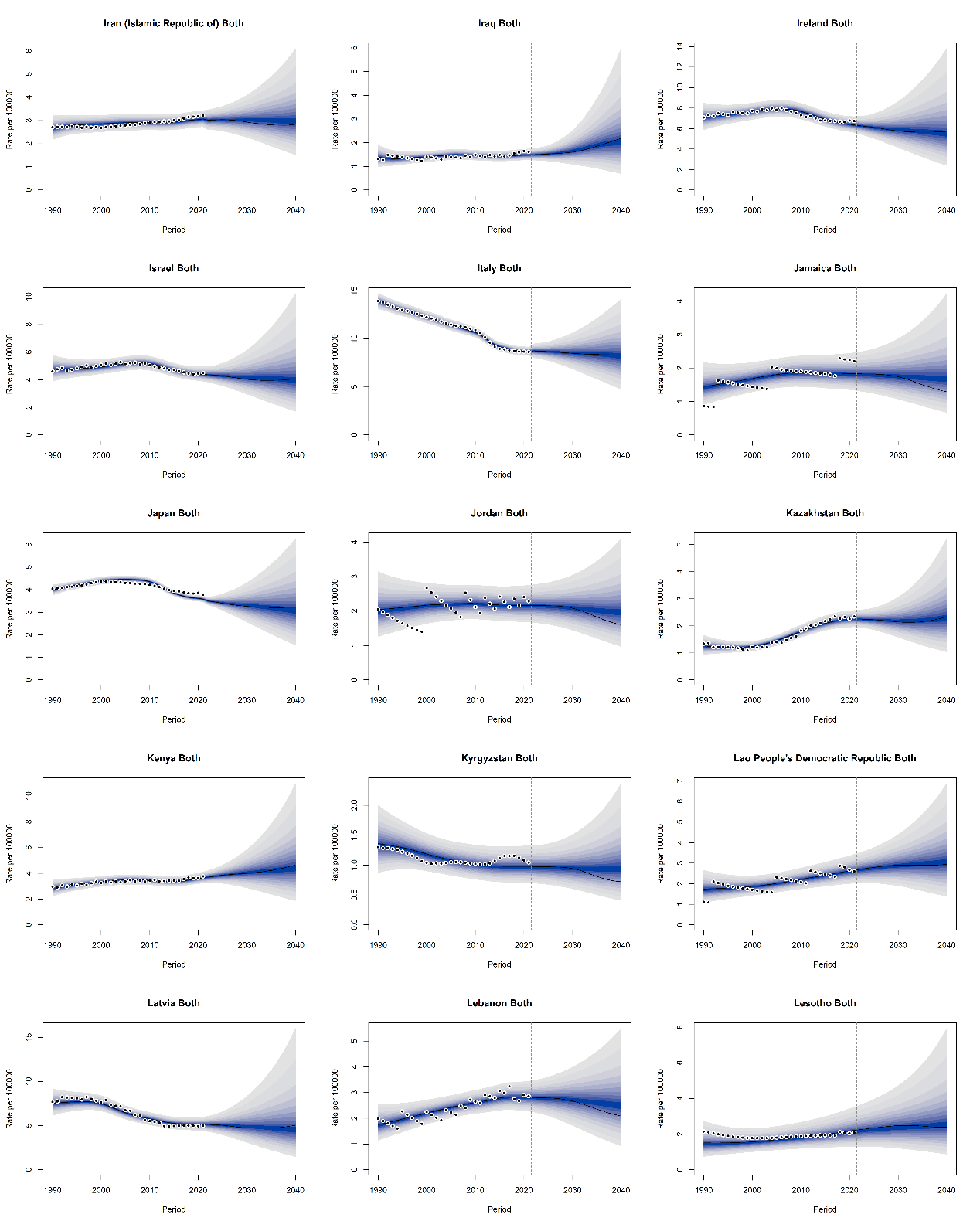
**

**
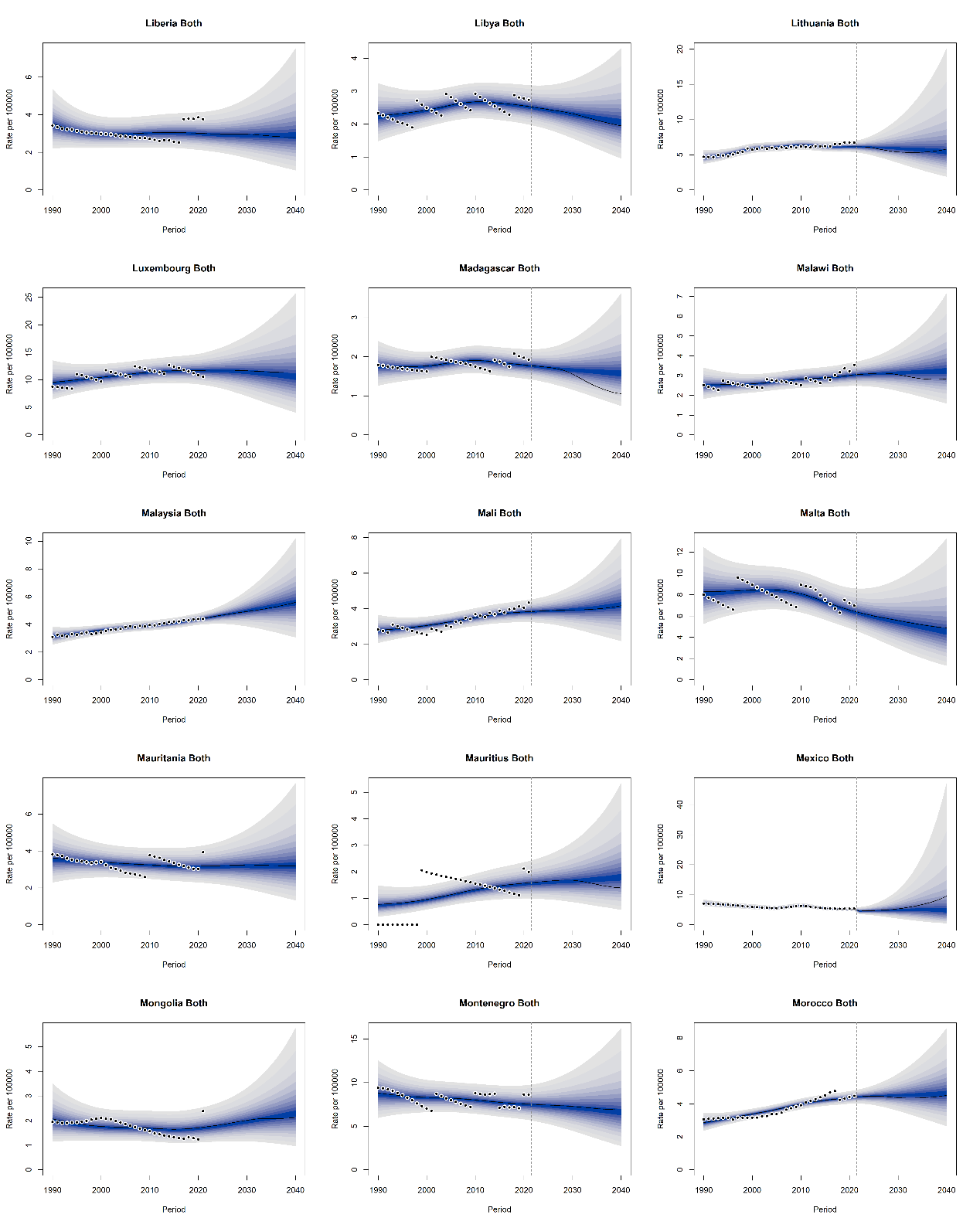
**

**
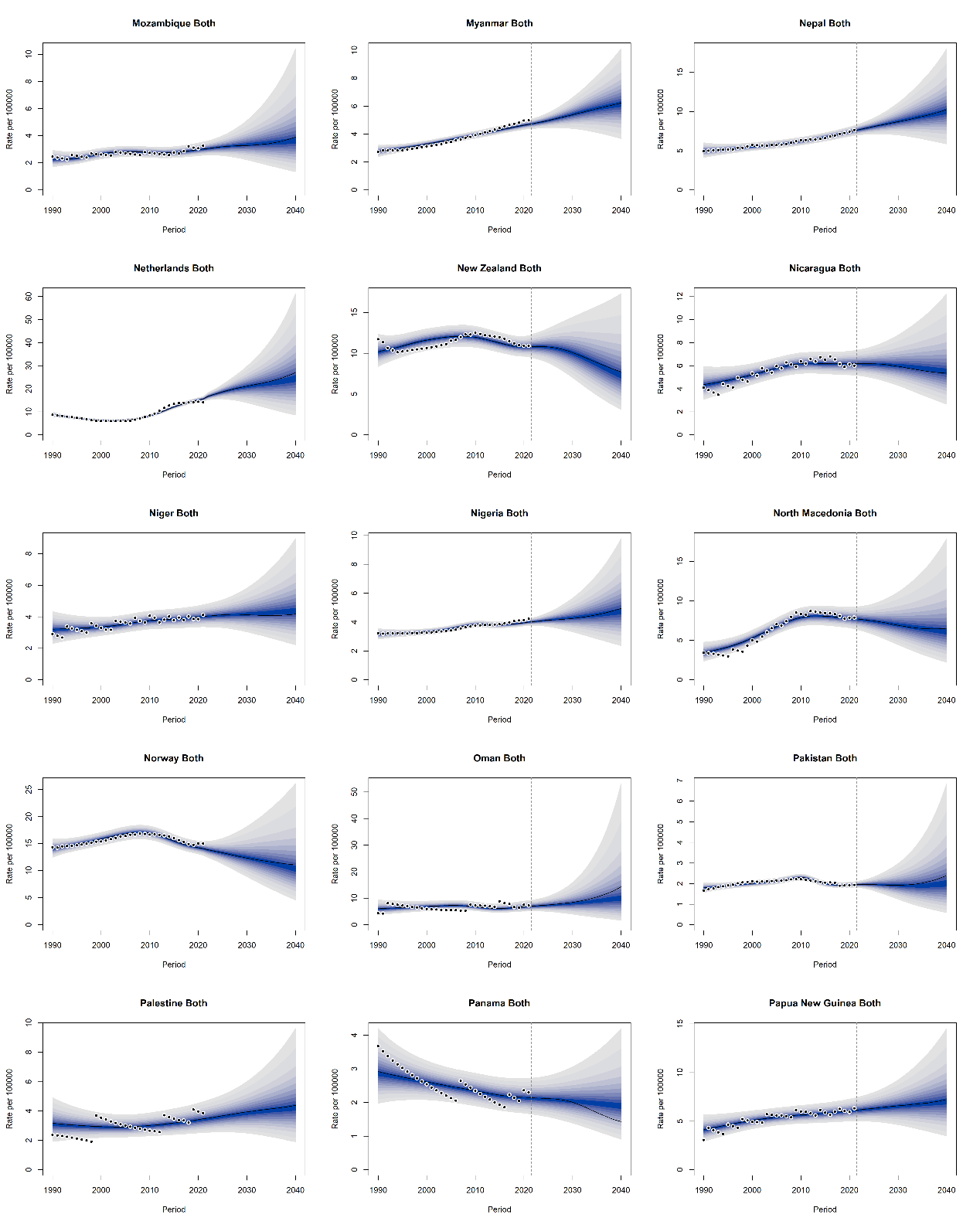
**

**
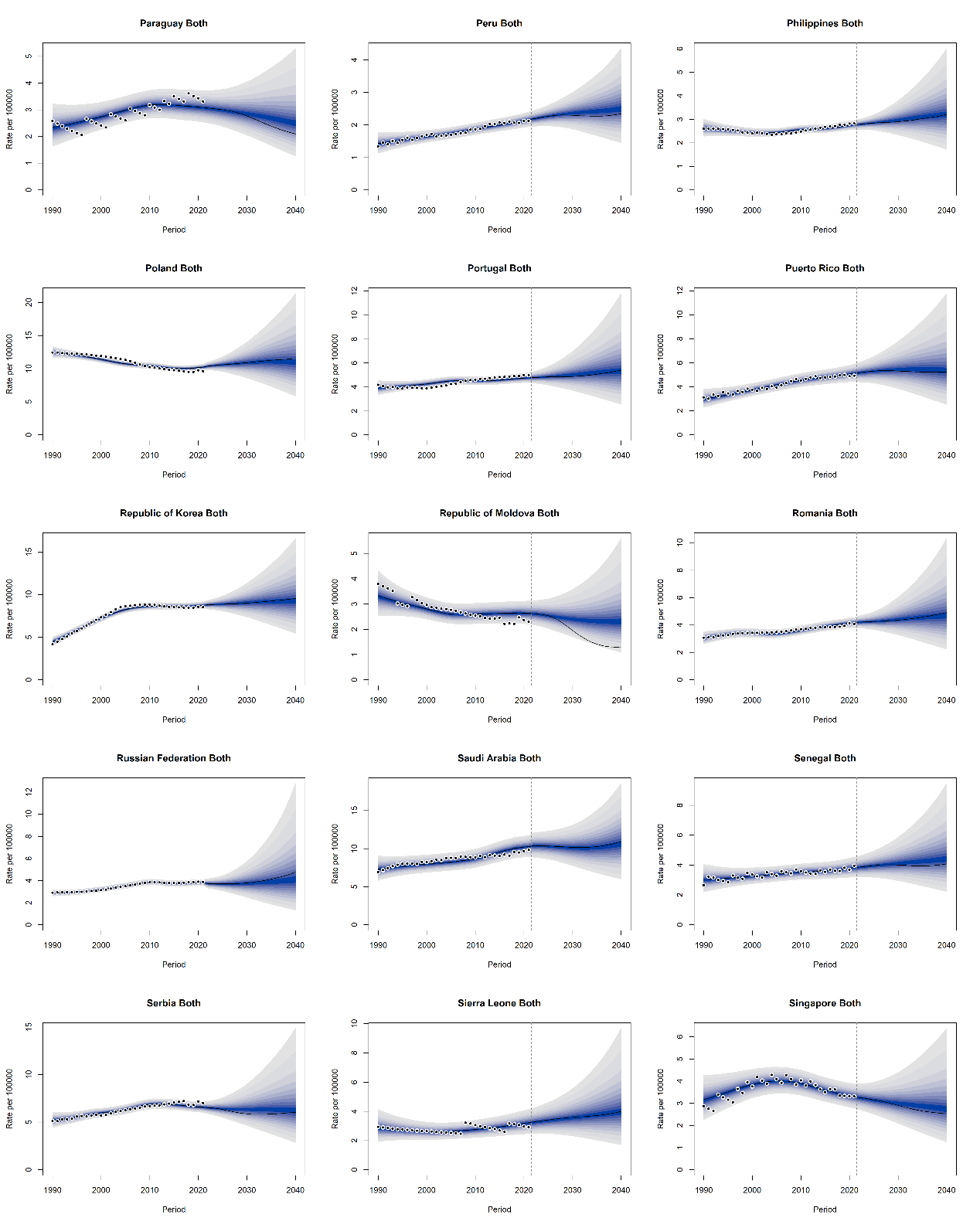
**

**
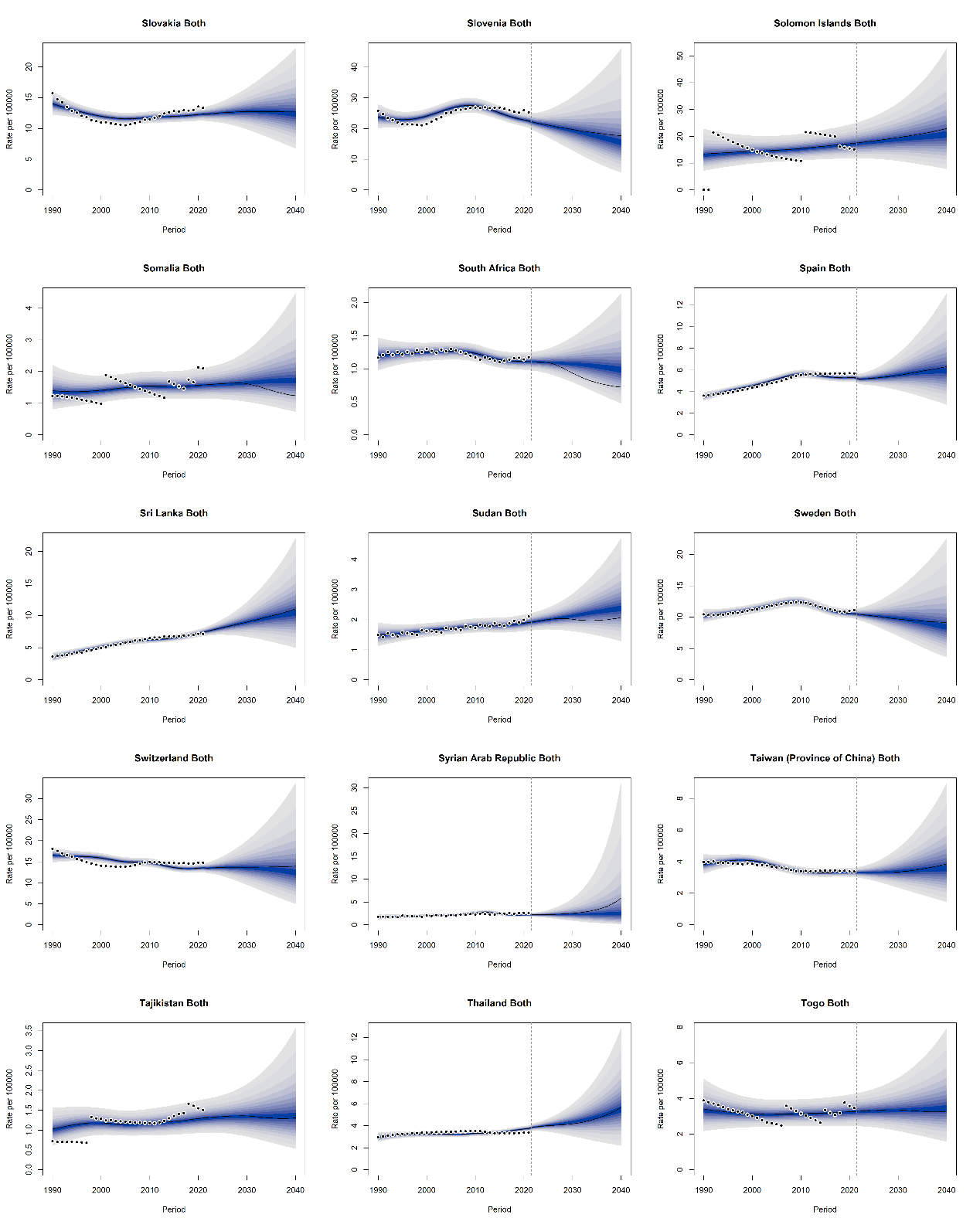
**

**
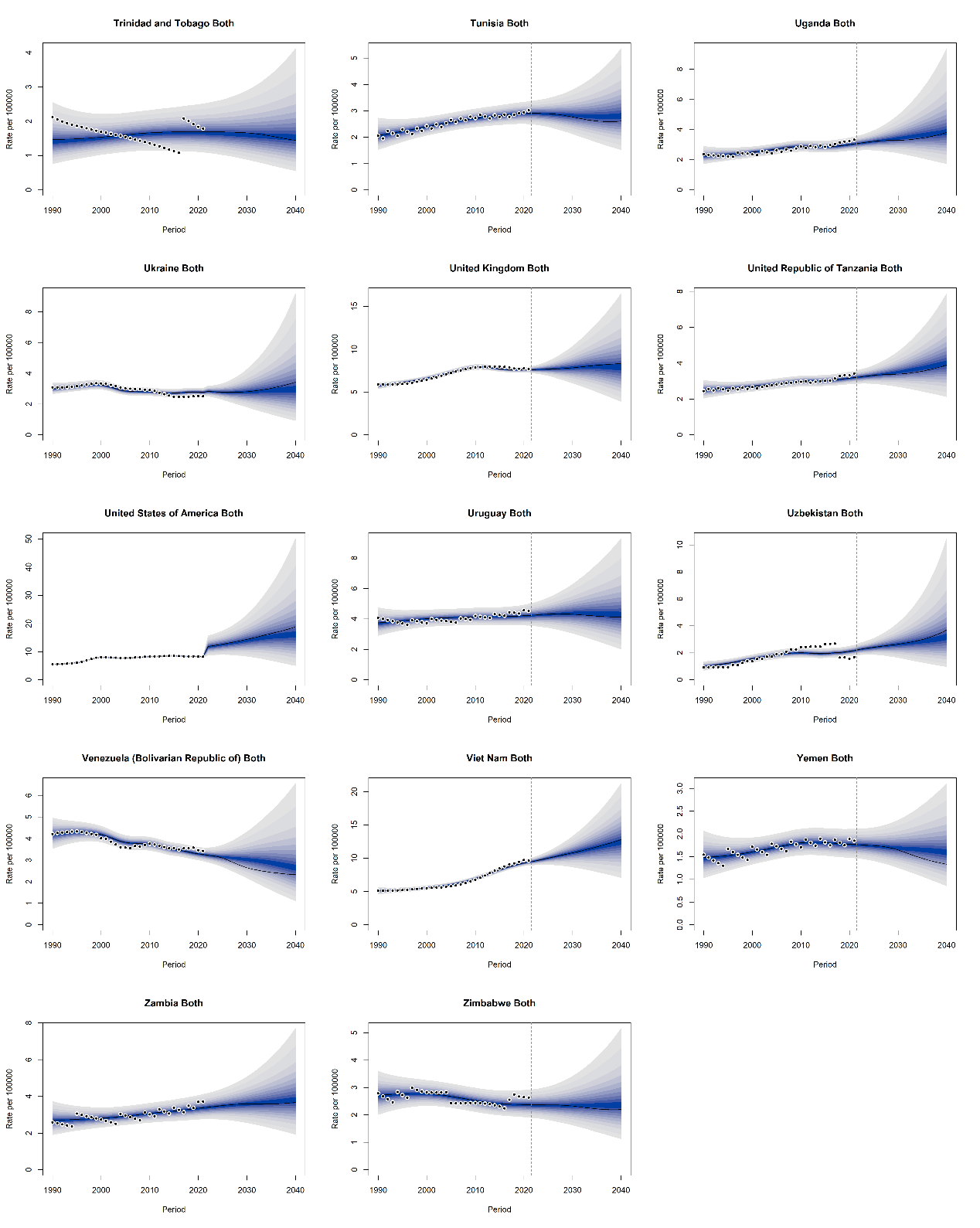
**

Rates denote age-standardized rates.
